# Mapping the Financial Risk of Iran’s Pharmaceutical System: A Cross-sectoral Qualitative Study

**DOI:** 10.5812/ijpr-170273

**Published:** 2026-08-19

**Authors:** Paniz Zadehsoleyman, Mohammad Peikanpour, Hamid Pourasghari, Bahador Nayebi

**Affiliations:** 1 Department of Pharmacoeconomics and Pharma Management, School of Pharmacy, Shahid Beheshti University of Medical Sciences, Tehran, Iran; 2 Hospital Management Research Center, Iran University of Medical Sciences, Tehran, Iran; 3 Department of Health Economics, School of Pharmacy, Tehran University of Medical Sciences, Tehran, Iran

**Keywords:** Financial Risk Management, Pharmaceutical System, Liquidity Risk, Credit Risk, Market Risk, Qualitative Study

## Abstract

**Background:**

Financial risk in pharmaceutical systems extends beyond supply-chain interruptions and is shaped by macroeconomic, regulatory, and governance dynamics. In Iran, where the pharmaceutical sector is characterized by structural dependence on imports, fragmented governance, administered pricing, and persistent exposure to macro-financial volatility, evidence on how these risks are generated, transmitted, and managed across institutional domains remains limited.

**Objectives:**

This study aimed to provide a cross-sectoral mapping of key financial risks in Iran’s pharmaceutical system and to examine the mechanisms through which these risks affect firms’ stability and national medicine security.

**Methods:**

An exploratory qualitative design was used, based on semi-structured interviews with 35 experts from regulatory and policy bodies, financial and insurance institutions, pharmaceutical manufacturers and distributors, academic institutions, and commercial stakeholders. Data were analyzed using thematic analysis.

**Results:**

Thematic analysis identified four analytical dimensions—drivers, transmission channels, consequences, and control responses—across five interrelated financial-risk categories: liquidity risk, market risk, credit risk, political risk, and operational risk. Risks were described as mutually reinforcing through liquidity constraints, pricing instability, macro-financial volatility, informal credit allocation, implementation weaknesses, and sanctions-related pressures, thereby generating systemic effects across the pharmaceutical sector. The study contributes a novel cross-sectoral framework for understanding how macroeconomic, regulatory, financial, and governance-related risks interact to reinforce systemic financial instability in pharmaceutical systems.

**Conclusions:**

Financial risk in Iran’s pharmaceutical sector is a system-level phenomenon arising from the interaction of macroeconomic instability, fragmented governance, and structural dependencies. Strengthening financial resilience will require integrated reforms in public financial management, pricing and credit governance, market-stabilization mechanisms, and depoliticized regulatory decision-making to safeguard both sectoral sustainability and national medicine security.

## 1. Background

Financial risk management is recognized as a core component of health-system performance; however, in pharmaceutical systems, it is more complex because it is shaped by interactions among pricing, regulation, reimbursement delays, and macroeconomic instability (1). Financial risk in pharmaceutical systems is not limited to firm-level profitability or financial performance; rather, it arises from interactions among pricing regulation, reimbursement mechanisms, supply-chain dependencies, and macroeconomic conditions. In such contexts, weaknesses in financial governance and coordination can amplify existing risks and generate system-wide instability (2). Accordingly, healthcare supply chains are increasingly evaluated using key performance indicators such as reliability, responsiveness, agility, and cost efficiency to assess risk exposure and resilience (3). Rising healthcare and pharmaceutical expenditures have intensified financial pressure on health systems, increasing the importance of financial risk management in the pharmaceutical sector (1).

In many developing countries, medicines account for 20 - 60% of total health spending, highlighting their substantial financial burden on both households and government health budgets and underscoring the strategic importance of pharmaceutical financing and procurement systems (4). However, organizational performance within the pharmaceutical sector is influenced by variations in governance structures, managerial capabilities, and organizational context, leading to substantial differences in firm performance (5).

Weak public financial management systems can generate and amplify financial risks by disrupting budgeting cycles, delaying payments, and weakening financial coordination. Evidence from South Africa illustrates how such weaknesses contribute to systemic financial instability in health systems (6). Previous studies across healthcare systems suggest that weak financial governance, fragmented coordination, and unmanaged cost pressures increase institutional financial vulnerability and reduce organizational resilience (7-11).

Pharmaceutical supply chains are highly exposed to financial risks, particularly through demand volatility, weak coordination mechanisms, and credit constraints. These factors disrupt cash-flow cycles, increase receivable periods, and generate liquidity pressures across supply-chain actors, ultimately reducing system resilience and amplifying financial vulnerability (12-15). Within the pharmaceutical sector, complementary evidence points to similarly pervasive systemic vulnerabilities. Evidence from Iran shows that the pharmaceutical supply chain faces deep structural vulnerabilities, with studies identifying financial and economic risks as dominant exposures alongside systemic challenges in financing, information systems, insurance mechanisms, and governance capacity (16, 17). Across developing-country settings, studies consistently report that weak pricing governance, financing constraints, and supply-chain fragmentation undermine financial resilience and operational continuity in pharmaceutical systems.

These operational and supply-chain vulnerabilities are closely linked to broader financial risks, particularly liquidity constraints and credit-related pressures that affect firms’ continuity and resilience.

Credit risk represents a major financial exposure in pharmaceutical systems, particularly in settings with weak credit governance and reliance on relationship-based financing. Such conditions increase default risk and create instability across supply-chain actors (18-20).

Market-related financial risks constitute another core component of the broader financial-risk landscape in the pharmaceutical industry.

Market-related financial risks in pharmaceutical systems arise from regulated pricing, demand uncertainty, and exposure to macroeconomic factors such as exchange-rate and interest-rate fluctuations. In developing-country contexts, weak or inconsistently implemented pricing policies can create price distortions, misalign costs and revenues, and generate financial instability for pharmaceutical firms (19-21).

Firm-level evidence also highlights the importance of liquidity management, showing that inefficient or excessive liquidity does not necessarily improve financial performance in pharmaceutical companies (20, 22). In Iran, these financial risks are particularly pronounced due to the structural characteristics of the pharmaceutical system. The sector is characterized by administered pricing, heavy dependence on imported raw materials, exposure to exchange-rate volatility, and persistent delays in reimbursement from insurance organizations. In addition, fragmented governance structures, regulatory uncertainty, and constrained access to formal financing mechanisms further intensify financial pressures. These conditions transform firm-level financial challenges into system-wide vulnerabilities, directly affecting production continuity and access to essential medicines.

Taken together, existing evidence reveals a fragmented and siloed understanding of financial risks across the pharmaceutical landscape. Prior studies have largely examined isolated domains—such as supply-chain disruptions, market volatility, credit exposures, or liquidity constraints—without articulating how these risks interact across institutional, regulatory, and financial subsystems. Moreover, no research has offered a cross-sectoral perspective on how financial risks originate, propagate, and accumulate within Iran’s pharmaceutical system, despite its dependence on imported inputs, exposure to macroeconomic volatility, and structurally constrained financing environment. This gap limits policymakers’ ability to anticipate systemic vulnerabilities and design coordinated, evidence-informed responses.

## 2. Objectives

Accordingly, this study adopted a cross-sectoral, exploratory approach to clarify how financial risks are perceived, transmitted, and managed across institutional domains in Iran’s pharmaceutical sector and to generate insights that may support more coherent and resilient financial risk management strategies.

## 3. Methods

This study employed an exploratory qualitative design to examine the dimensions and mechanisms of financial risk management in Iran’s pharmaceutical system from an intersectoral perspective. Purposive and snowball expert sampling were used to identify participants with at least 5 years of professional experience in pharmaceutical policy, financial management, supply-chain operations, or macroeconomic regulation, either within or outside the pharmaceutical sector (23). The inclusion criteria emphasized demonstrable expertise in pharmaceutical financial systems and familiarity with systemic financial challenges.

Initial participants were identified purposively through consultation with academic supervisors and review of key institutional and professional positions relevant to pharmaceutical policy, financing, regulation, insurance, industry, distribution, academia, and healthcare delivery. Snowball sampling was then conducted by asking interviewed participants to identify other eligible experts with relevant knowledge and experience. Suggested participants were screened against the inclusion criteria before invitation.

Potential participants were contacted by telephone and text message and voluntarily invited to participate in face-to-face semi-structured interviews. A total of 35 experts were recruited and interviewed between July 2024 and July 2025. Interviews lasted 45 to 120 minutes and followed an interview guide comprising open-ended questions. A summary of the semi-structured interview guide and sample interview questions is provided in Appendix 2 in the Supplementary File. Data collection and analysis were conducted concurrently, and interviews continued until thematic saturation was achieved, with no substantially new themes or concepts emerging in later interviews. Verbal informed consent was obtained from all participants before data collection. No participants withdrew after agreeing to participate.

The 5-category financial-risk framework was derived from the literature and used to guide data collection. The framework included liquidity, market, credit, operational, and political risks and informed the development of the interview guide and discussion topics (Figure 1). Although these predefined categories structured data collection, thematic analysis remained inductive. Initial coding was data-driven, and themes were developed iteratively from participants’ narratives rather than being imposed a priori by the conceptual framework.

**Figure 1.**
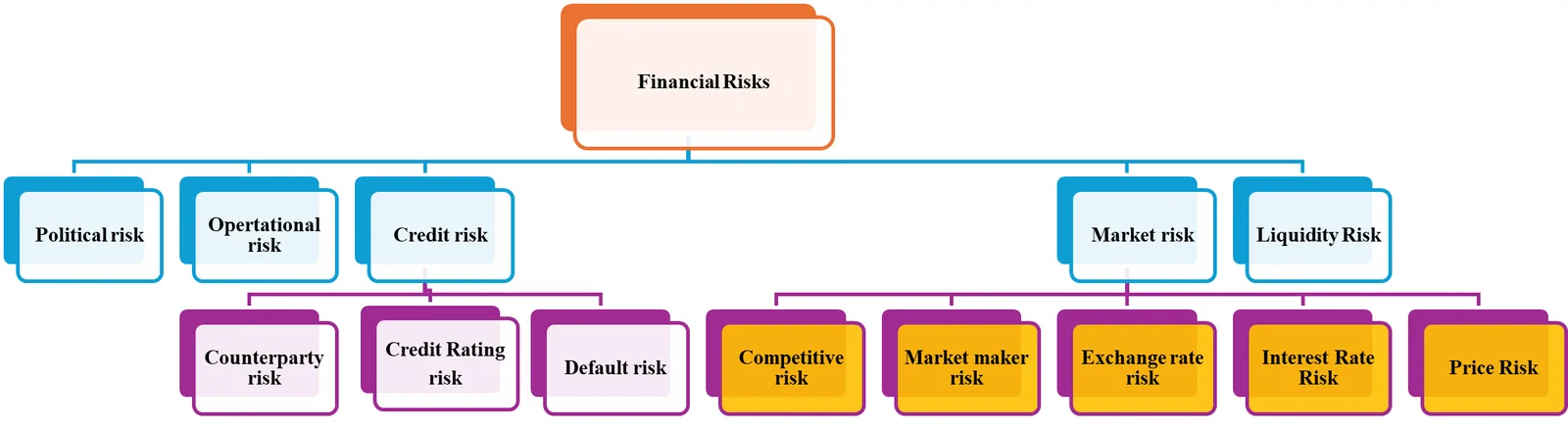
Classification of financial-risk categories and their subdimensions in the pharmaceutical system.

Data were analyzed using thematic analysis. Interview transcripts were coded in MAXQDA 2022 using meaning-based coding units. Initial codes and themes were independently reviewed by 2 researchers. Differences in coding and interpretation were discussed until agreement was reached, and unresolved discrepancies were reviewed by a third researcher. Through repeated cycles of coding, categorization, and conceptual refinement, recurrent patterns and cross-sectoral relationships shaping financial-risk management in Iran’s pharmaceutical system were identified and organized into themes. Appendix 1 in the Supplementary File provides the Consolidated Criteria for Reporting Qualitative Research (COREQ) checklist and supporting methodological information.

## 4. Results

A total of 35 semi-structured interviews were conducted with experts from a wide spectrum of organizations, including the Ministry of Health and Medical Education, the Iran Food and Drug Administration, the Plan and Budget Organization, the Ministry of Economic Affairs and Finance, the Central Bank of Iran, the Supreme Council of Health Insurance, the Competition Council, the Innovation and Prosperity Fund, the Health Insurance Organization, and representatives from the pharmaceutical industry, distribution companies, universities, and the Chamber of Commerce.

This diverse participant composition provided an intersectoral perspective by capturing insights from regulatory, financial, insurance, industrial, and service-delivery domains relevant to financial-risk management in Iran’s pharmaceutical system (Appendix 1 in the Supplementary File for additional methodological and participant details).

Key characteristics of the interviewees are summarized in Table 1.

**Table 1.** Characteristics of the Interviewees

Variables	Values
**Average Number of Interview Words**	5500
**Average Interview Time (min)**	70
Education	
Ph.D.	22
Pharm.D.	8
M.D.	1
Bachelor of Laws	1
**Average Age (y); (Range)**	50 (34 - 72)
**Gender**	
Male	28
Female	7
**Number Interviewed**	35

A total of 1,006 initial codes were generated from the interview data. These codes, encompassing all identified risk categories, were subsequently organized into 4 overarching analytical dimensions: policy and environmental drivers, transmission channels to firms, consequences, and control responses. The analysis identified several key financial risks, which are presented in the following sections with their corresponding themes. A comprehensive list of all extracted codes is provided in Appendix 3.

### 4.1. Liquidity Risk

Liquidity risk was identified as a major financial vulnerability in Iran’s pharmaceutical sector. Key drivers included delayed reimbursements from insurance organizations, extended receivable cycles, dependence on imported raw materials, and inflation-related cost pressures, all of which constrained firms’ cash flow and their ability to meet short-term obligations.

As one financial manager explained:

“Insurers pay months late, but foreign suppliers require immediate payment. This mismatch creates severe pressure on our liquidity.” (P15, Insurance and Reimbursement Sector)

Participants also highlighted the role of inventory practices. Maintaining large safety stocks to avoid drug shortages was reported as a common strategy; however, it ties up substantial working capital. As a production manager noted:

“We keep high inventory levels to avoid shortages, but this ties up our cash.” (P14, Pharmaceutical Distribution and Supply Chain Sector)

These liquidity constraints frequently resulted in production delays, postponed investments, and increased financial fragility. Firms often relied on factoring, advance payments, or renegotiated terms to bridge cash-flow gaps, although these measures increased financing costs and provided only temporary relief.

Liquidity constraints were associated with production delays, postponed investments, and financial pressure on firms. To manage these conditions, participants described strategies including factoring, advance payments, renegotiation of payment terms, and short-term financing instruments. Liquidity risk was organized across 4 analytical dimensions: drivers and policy environment, transmission channels to firms, consequences, and control responses. These dimensions are presented in Figure 2.

**Figure 2.**
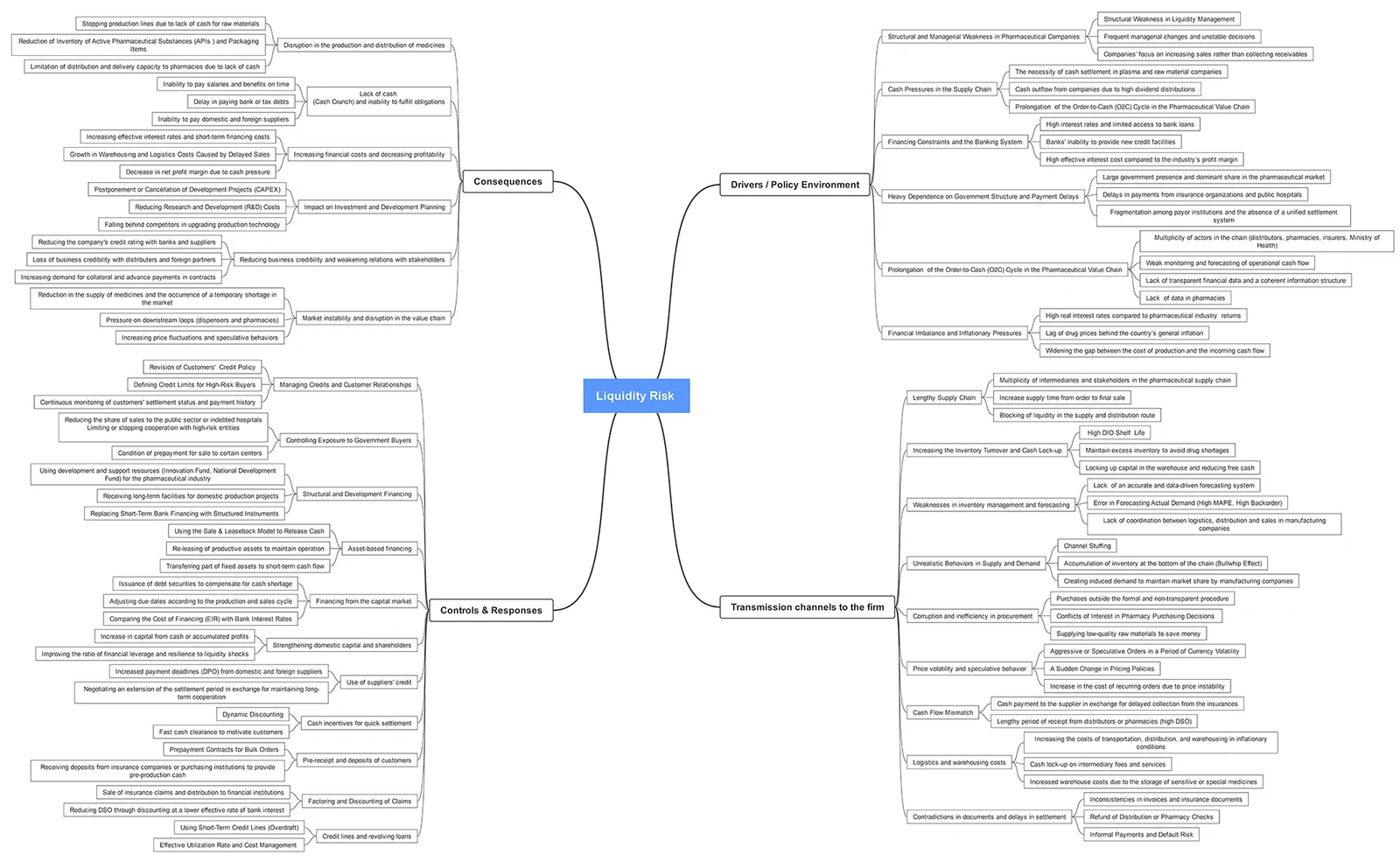
Analytical dimensions of liquidity risk in Iran’s pharmaceutical system.

### 4.2. Market Risk in the Pharmaceutical Industry

#### 4.2.1. Pricing Risk

Pricing risk was identified as a key dimension of market risk in Iran’s pharmaceutical sector. Participants reported that centrally administered pricing limits firms’ ability to adjust prices in line with production costs. As one senior financial manager remarked:

“We import raw materials at the free-market exchange-rate, but the government dictates the selling price. Sometimes the approved price is so low that we are literally manufacturing at a loss.” (P5, Pharmaceutical Industry and Investment Sector)

Participants also described a lack of transparency and predictability in the pricing system. Sudden pricing revisions and limited stakeholder consultation were reported to create uncertainty in production planning. These conditions were associated with reduced margins, liquidity pressures, and changes in firm behavior, including adjustments in product portfolios and production decisions. Some participants noted a shift away from low-margin products and increased reliance on imports.

Pricing risk was organized across four analytical dimensions: drivers and policy environment, transmission channels to firms, consequences, and control responses. A detailed representation of these dimensions is presented in Figure 3.

**Figure 3.**
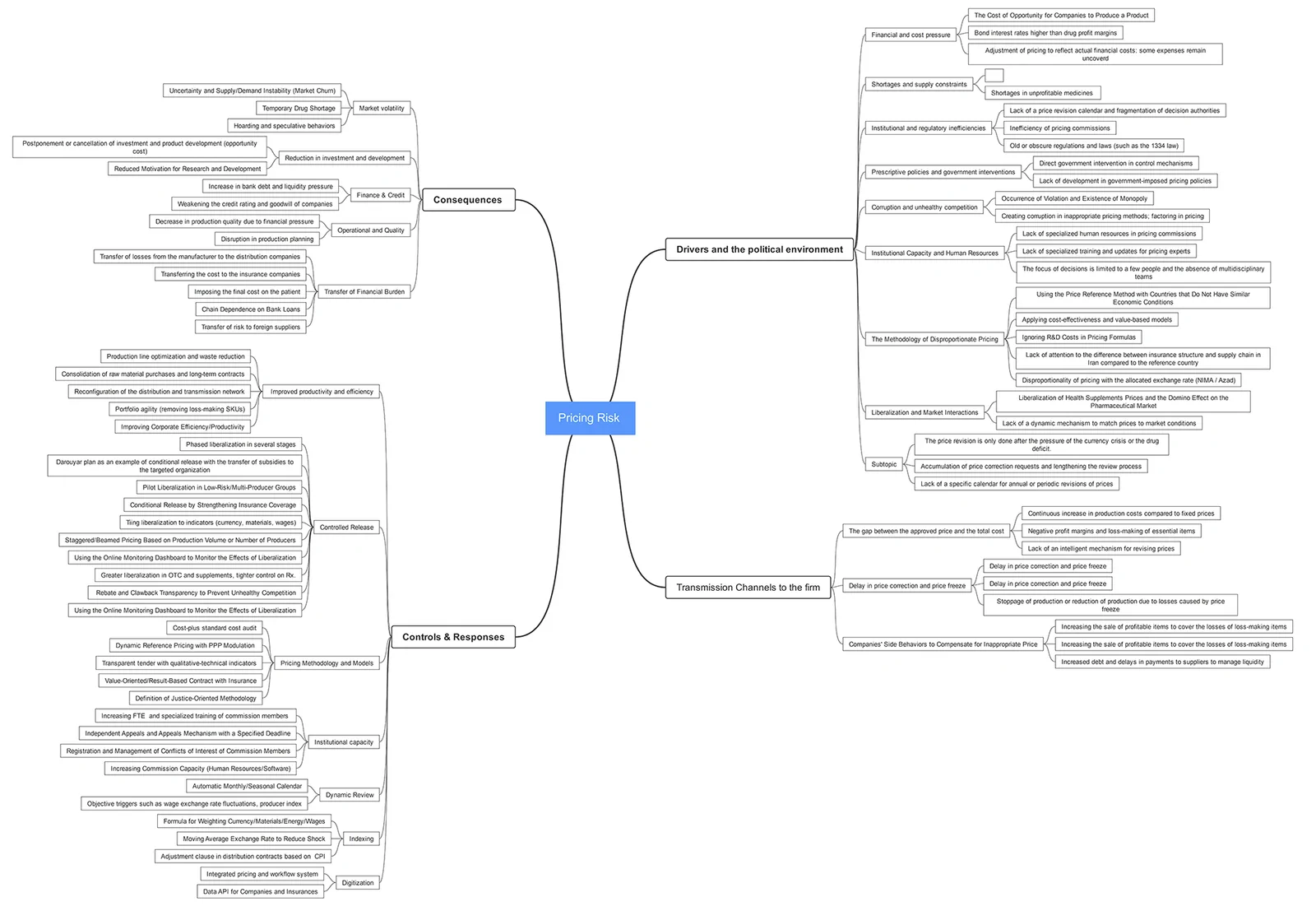
Analytical dimensions of pricing risk in Iran’s pharmaceutical system.

#### 4.2.2. Interest-Rate Risk

Interest-rate risk was identified as an important source of financial exposure in Iran’s pharmaceutical sector. Participants reported that a large share of working capital and investment activities is financed through bank loans, making firms sensitive to changes in lending rates.

Participants noted that elevated lending rates often undermine the economic feasibility of pharmaceutical projects. As one senior financial manager explained:

“When bank interest rates exceed the industry’s average profit margin, borrowing becomes irrational. We either halt development projects or rely on shareholder capital.” (P18, Financial and Investment Policy Sector)

Participants also highlighted the relationship between inflation and interest rates. Situations in which inflation exceeded financial returns were described as increasing financial pressure on firms and affecting investment decisions. Some participants reported reduced reliance on bank financing and increased use of internal resources or shareholder capital. These strategies were described as constrained and not always sustainable. Interest-rate risk was organized across four analytical dimensions: drivers and policy environment, transmission channels to firms, consequences, and control responses. A detailed representation of these dimensions is presented in Figure 4.

**Figure 4.**
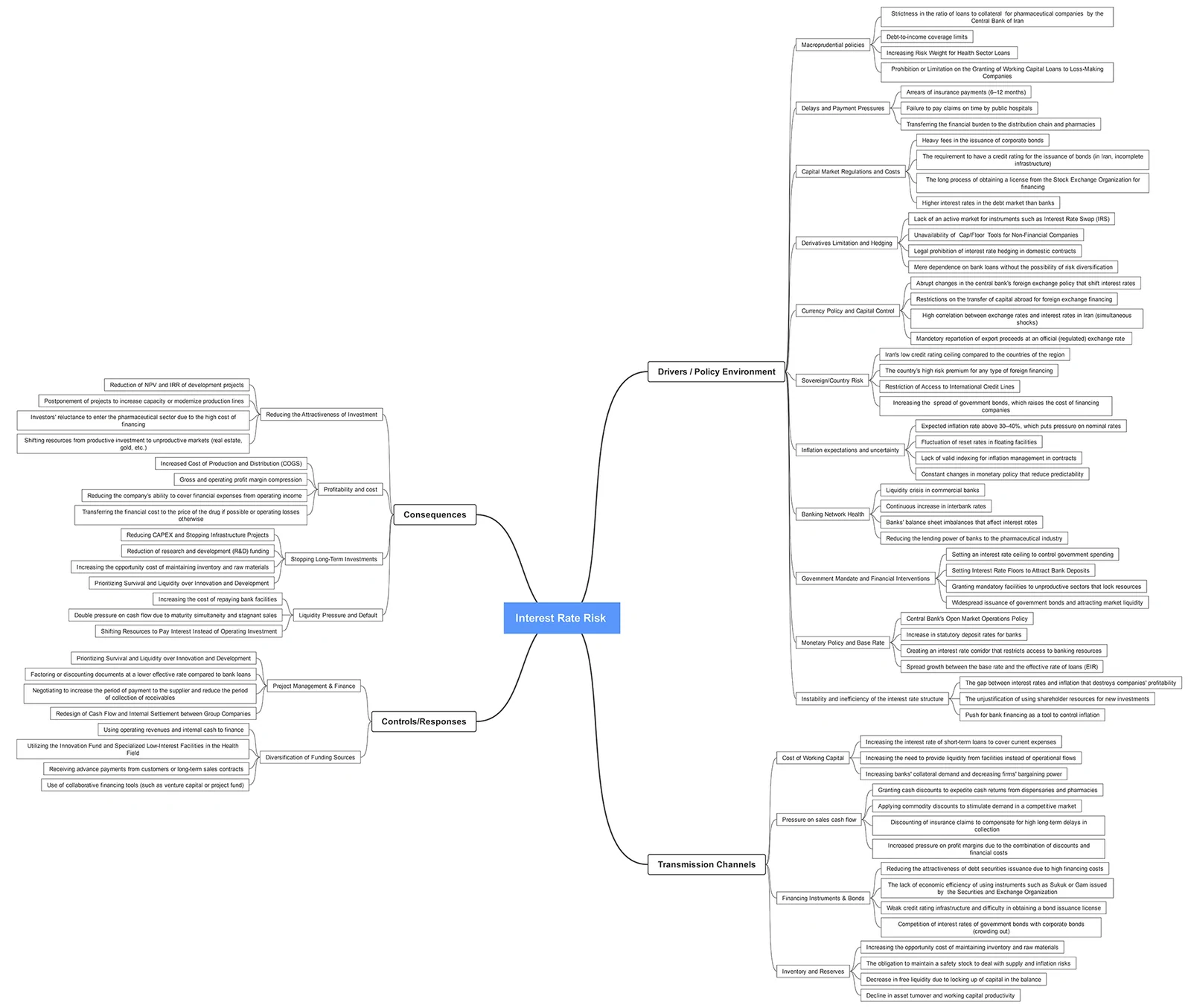
Analytical dimensions of interest-rate risk in Iran’s pharmaceutical system.

#### 4.2.3. Exchange-Rate Risk

Exchange-rate risk was identified as a major financial exposure in Iran’s pharmaceutical sector. Participants reported that a large share of pharmaceutical raw materials is imported, making production costs sensitive to currency fluctuations.

Interviewees consistently identified currency volatility and the presence of multiple exchange rates as major drivers of financial instability. As one financial manager noted:

“When the exchange rate changes constantly, no financial plan holds. We buy raw materials at one rate, but by the time the drug reaches the market everything has changed—our entire cost structure collapses.” (P4, Monetary and Exchange Policy Sector)

Participants also emphasized that the multitiered exchange-rate system generates market distortions and rent-seeking opportunities. As one senior executive explained: “With multiple exchange rates, firms with privileged access profit while others face losses. This destroys fair competition and makes costs completely unpredictable.” (P27, Public Health Budgeting Sector)

Delays in currency allocation and regulatory instability were also reported by participants, particularly in relation to procurement processes and supply continuity. Exchange-rate risk was organized across four analytical dimensions: drivers and policy environment, transmission channels to firms, consequences, and control responses. A detailed representation of these dimensions is presented in Figure 5.

**Figure 5.**
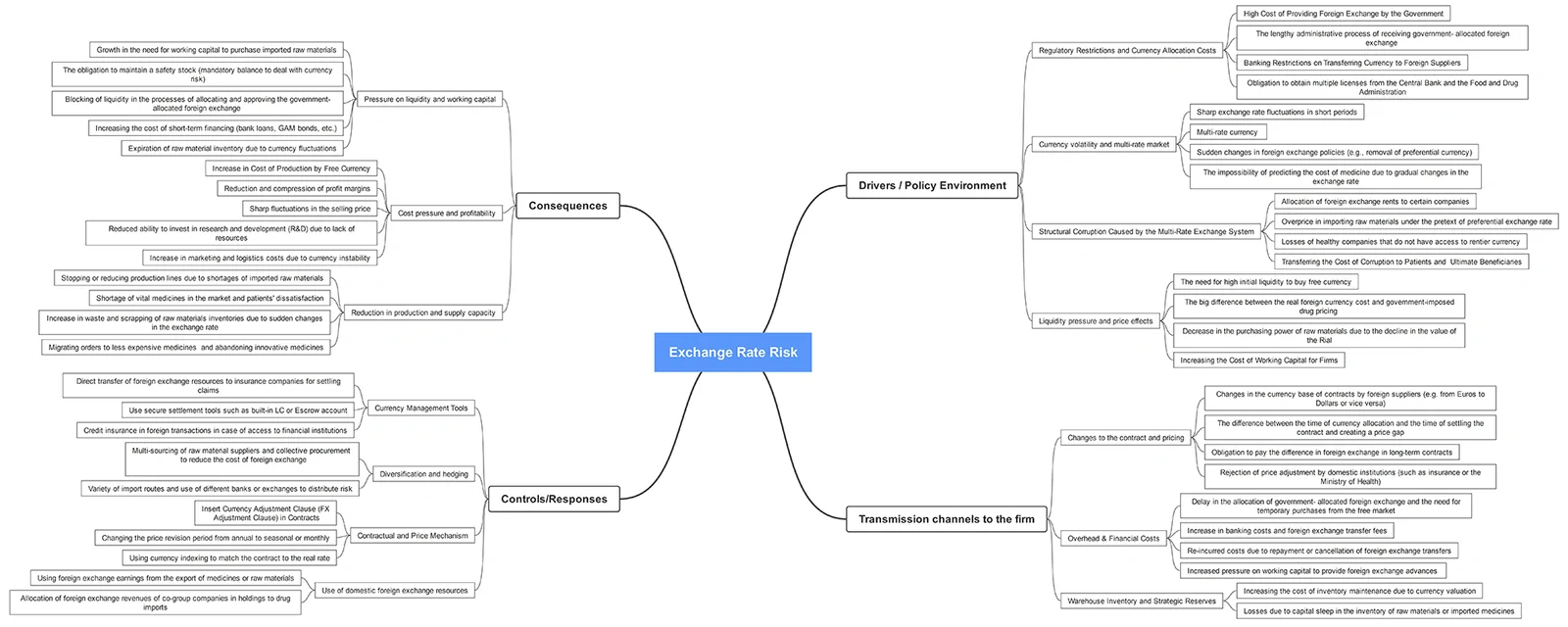
Analytical dimensions of exchange-rate risk in Iran’s pharmaceutical system.

#### 4.2.4. Market-Maker Risk

Market-maker risk was identified as a financial vulnerability in Iran’s pharmaceutical sector. Participants reported the absence of a clear legal definition, regulatory framework, and financial infrastructure related to market-making functions. As one senior executive noted:

“The market-maker in Iran has no legal definition, no governing law, and no supervisory body. Without legal existence, it cannot be regulated or held accountable.” (P16, Pharmaceutical Market Regulation Sector)

Participants also described gaps in financial and regulatory instruments, including the limited availability of mechanisms such as credit lines, factoring, and clearing systems. These conditions were reported to be associated with payment delays, the accumulation of receivables, and liquidity challenges across the supply chain. Market-maker risk was organized across four analytical dimensions: drivers and policy environment, transmission channels to firms, consequences, and control responses. A detailed representation of these dimensions is presented in Figure 6.

**Figure 6.**
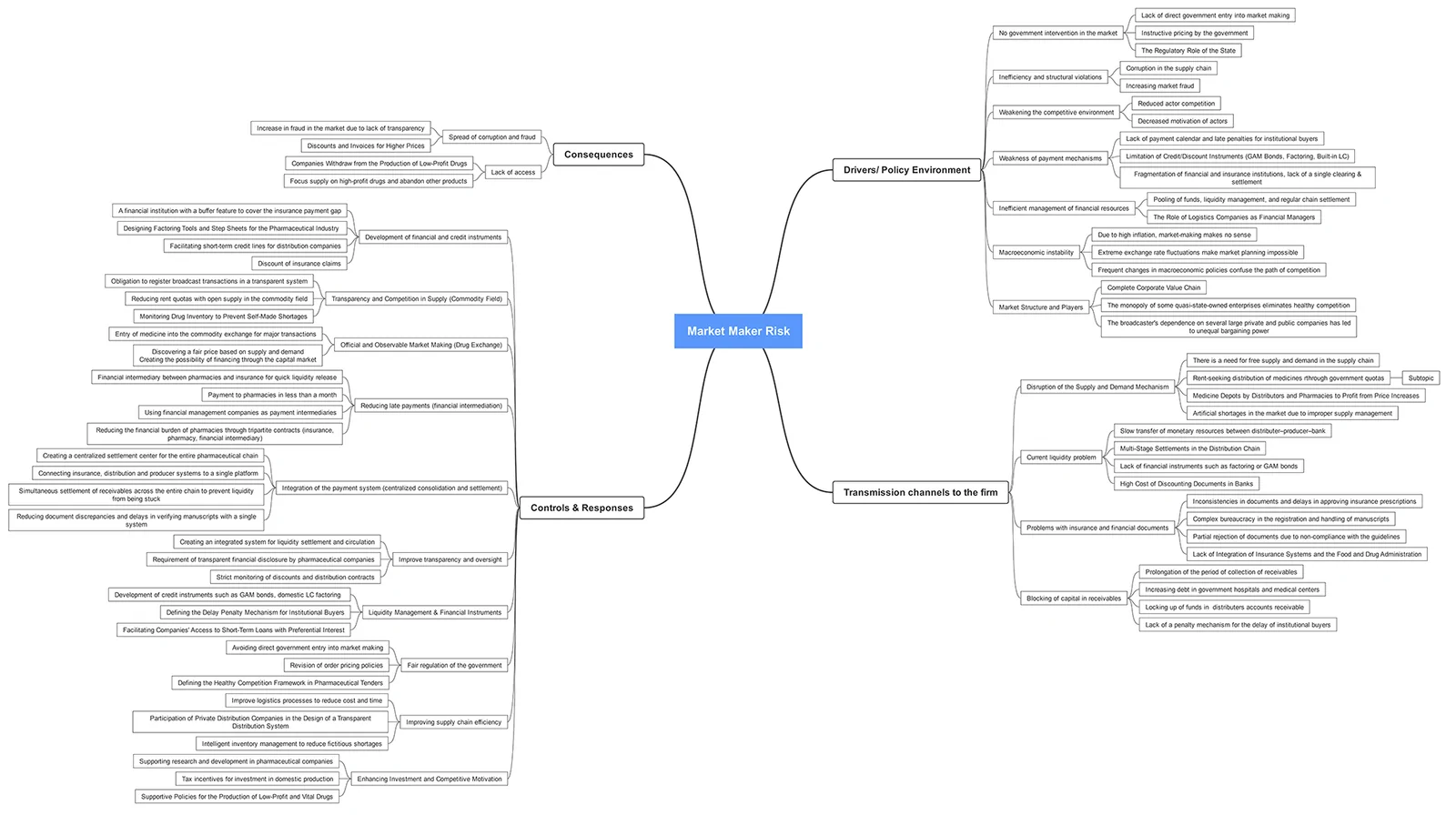
Analytical dimensions of market-maker risk in Iran’s pharmaceutical system.

#### 4.2.5. Competitive Risk

Competitive risk was identified as an important feature of Iran’s pharmaceutical sector. Participants reported that competitive conditions are influenced by regulatory arrangements, government intervention, and differences in access to resources. A central theme across interviews was the role of state-affiliated firms and differential access to foreign currency and regulatory support. As one financial manager stated:

“When some companies receive subsidized foreign currency or special support, there is no fair competition. We buy raw materials at the free-market rate and sell at controlled prices, while others profit from preferential treatment.” (P10, Central Bank of Iran)

Participants also described fragmented market structures and variations in firm capacity. The presence of imported and, in some cases, illicit products was reported to affect competitive conditions. These conditions were described as being associated with differences in cost structures, firm behavior, and market participation. Some participants referred to changes in product portfolios, pricing strategies, and market focus.

Competitive risk was organized across four analytical dimensions: drivers and policy environment, transmission channels to firms, consequences, and control responses. A detailed representation of these dimensions is presented in Figure 7.

**Figure 7.**
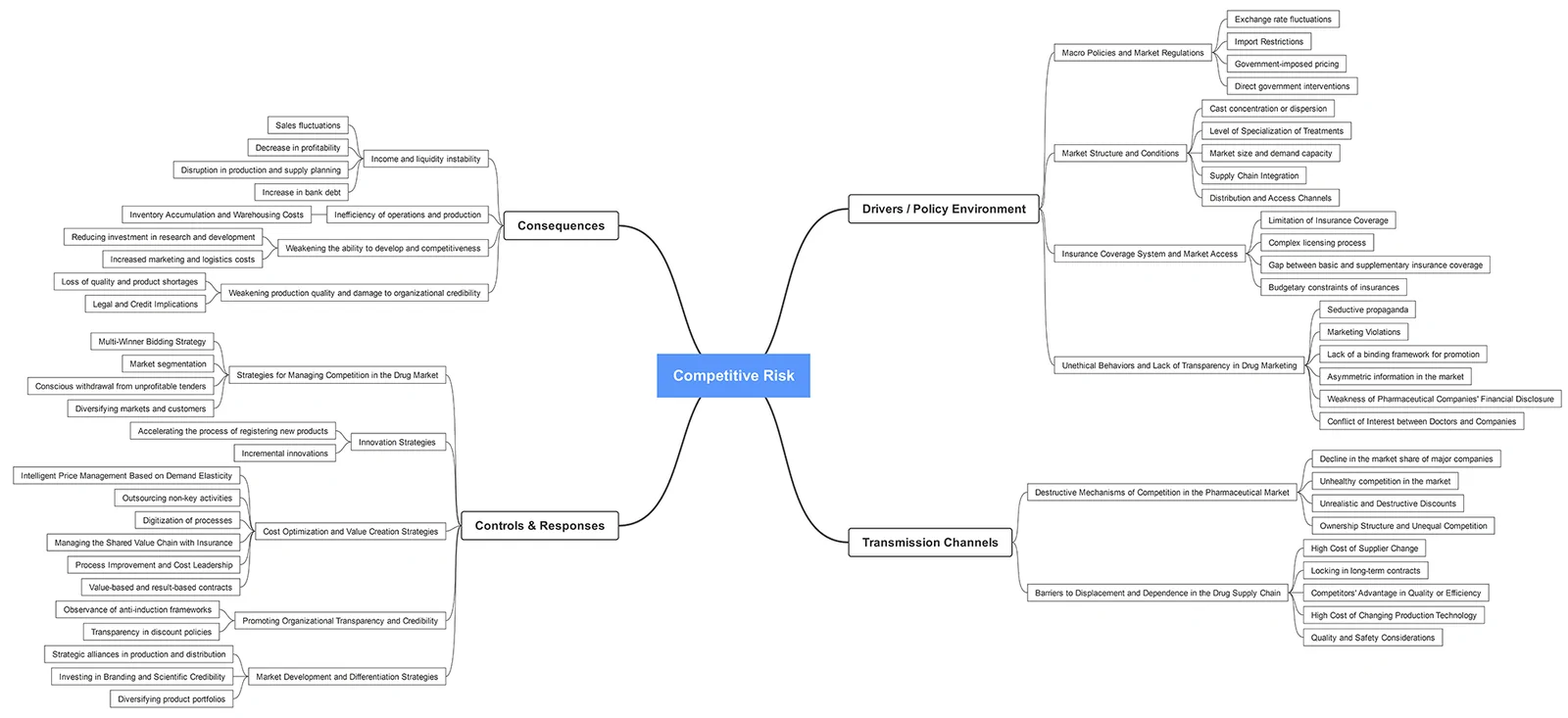
Analytical dimensions of competitive risk in Iran’s pharmaceutical system.

### 4.3. Credit Risk

#### 4.3.1. Default Risk

Default risk was identified as a financial exposure in Iran’s pharmaceutical sector. Participants reported several factors related to debt structure and financing conditions, including short-term borrowing, foreign-currency liabilities, and limited collateral. As one participant explained:

“When our income is in rials but our debts are in dollars, even a small currency shock makes it impossible to pay installments on time, and default risk rises immediately.” (P10, Central Bank of Iran)

Another recurring issue was the concentration of loan maturities within short periods, creating acute liquidity pressure. A financial manager described this dynamic as follows: “The main issue is the maturity wall—when several large loans mature at once, repayment becomes impossible unless we take on new debt, which only increases the risk.” (P8, Ministry of Economic Affairs and Finance)

Default risk was organized across four analytical dimensions: drivers and policy environment, transmission channels to firms, consequences, and control responses. A detailed representation of these dimensions is presented in Figure 8.

**Figure 8.**
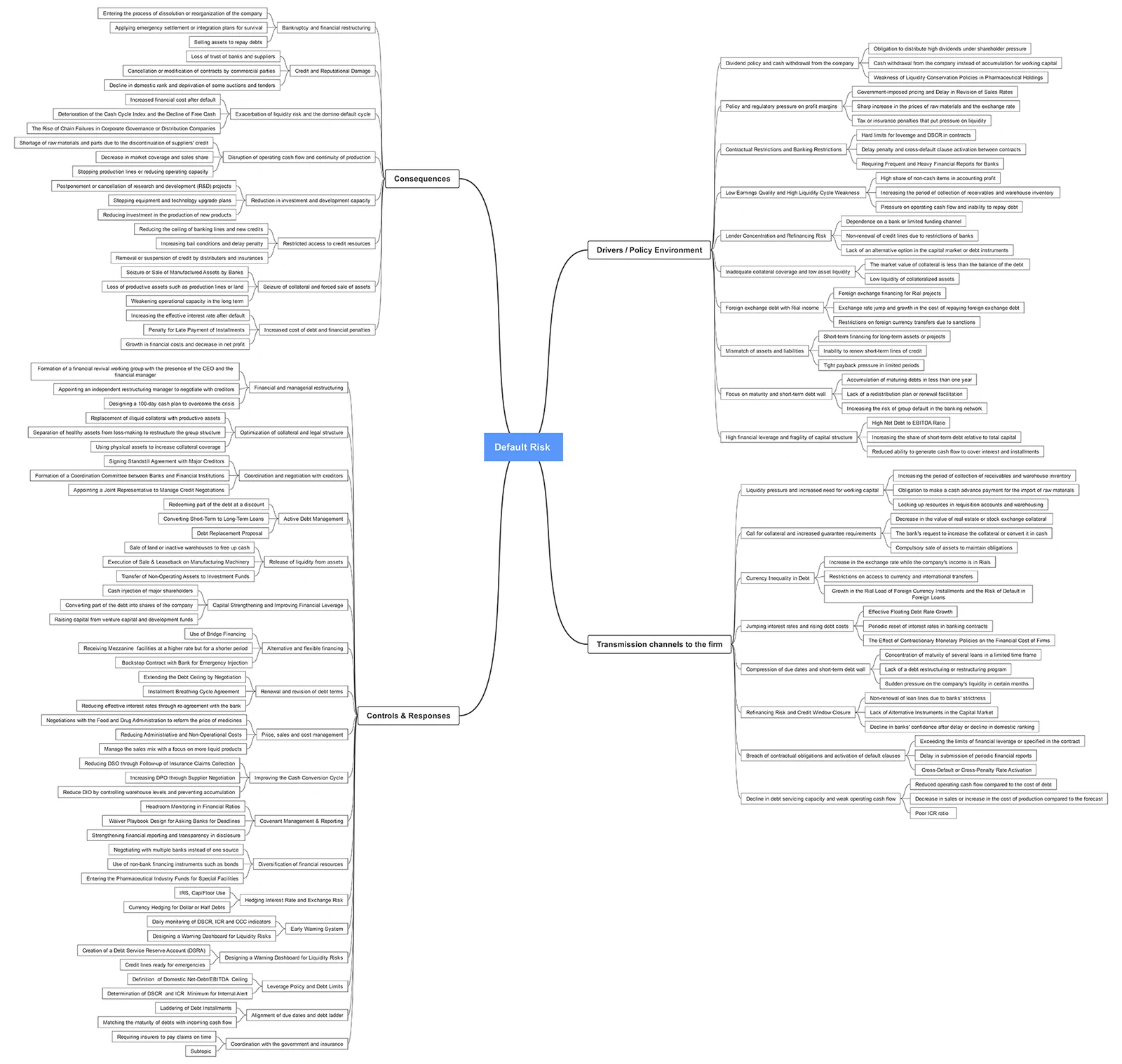
Analytical dimensions of default risk in Iran’s pharmaceutical system.

#### 4.3.2. Credit-Rating Change Risk

Credit-rating change risk was identified as a financial exposure in Iran’s pharmaceutical sector. Participants reported the absence of a formal and transparent credit-rating system and reliance on informal assessments.

As one financial manager noted:

“We don’t have a real credit-rating system. Banks decide based on personal connections, not financial performance.” (P18, Commercial Banking Sector)

Participants also described situations in which perceived financial difficulties, such as liquidity concerns, were associated with changes in financing conditions. These included stricter collateral requirements, shorter payment terms, and reduced access to credit. Some participants noted that financing conditions were not consistently linked to firm performance and that access to credit could vary across firms. Credit-rating change risk was organized across four analytical dimensions: drivers and policy environment, transmission channels to firms, consequences, and control responses. A detailed representation of these dimensions is presented in Figure 9.

**Figure 9.**
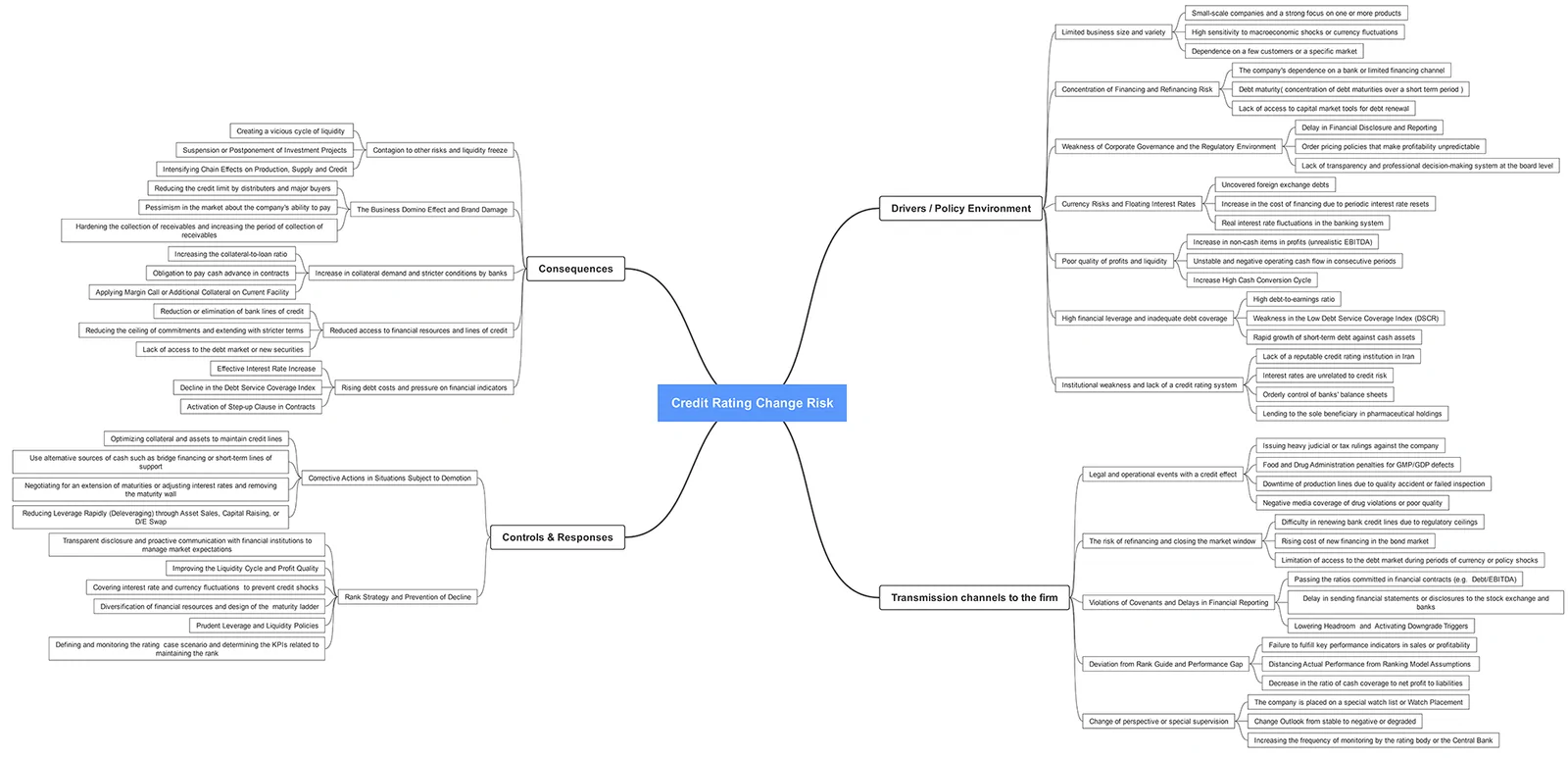
Analytical dimensions of credit-rating change risk in Iran’s pharmaceutical system.

#### 4.3.3. Counterparty Risk

Counterparty risk was identified as a financial exposure in Iran’s pharmaceutical sector. Participants reported delays in reimbursements from insurance organizations and the role of large distributors in shaping payment conditions. As one financial manager stated:

“Insurance organizations are the biggest source of counterparty risk. When they delay payments for months, the entire liquidity chain freezes.” (P20, Supreme Council of Health Insurance)

Participants also described the absence of transparent credit assessment systems and the use of relationship-based judgments in financial transactions. Some participants referred to challenges related to international transactions and intermediary arrangements.

These conditions were reported to be associated with cash-flow pressures, delays in production processes, and changes in contractual arrangements. Participants also described the use of strategies such as prepayments, collateral requirements, and selective engagement with partners.

Counterparty risk was organized across four analytical dimensions: drivers and policy environment, transmission channels to firms, consequences, and control responses. A detailed representation of these dimensions is presented in Figure 10.

**Figure 10.**
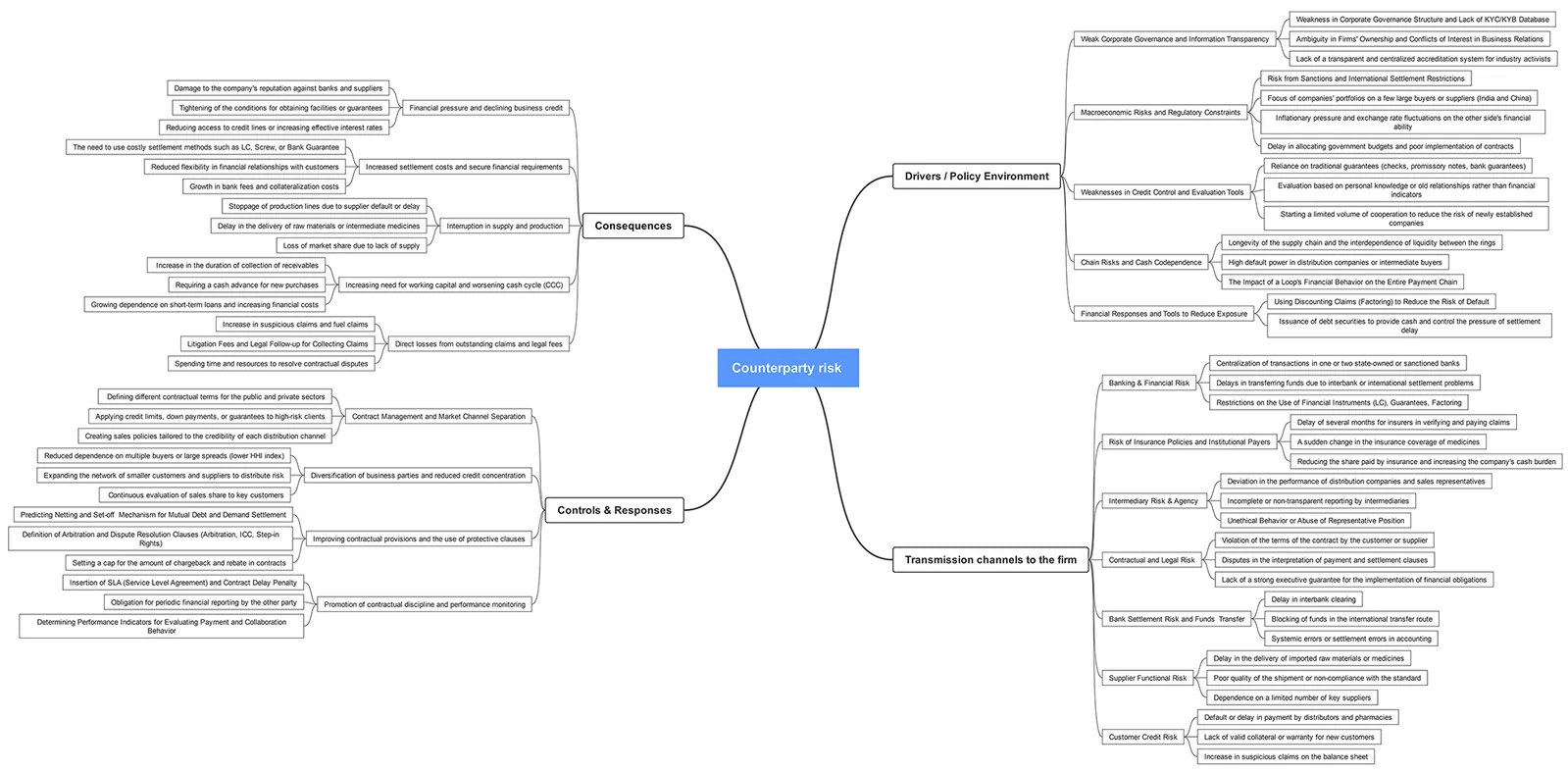
Analytical dimensions of counterparty risk in Iran’s pharmaceutical system.

### 4.4. Operational Risk

Operational risk emerged as a major systemic challenge in Iran’s pharmaceutical sector, driven by weak governance capacity, fragmented decision-making, and inconsistent policy environments. Although many firms develop formal strategies, outdated managerial systems and inadequate monitoring tools hinder effective implementation. As one production manager noted:

“We have strategies, but weak systems and lack of tools mean projects fail or face major delays.” (P6, Pharmaceutical Manufacturing Companies)

Frequent regulatory changes, bureaucratic bottlenecks, and overlapping institutional authorities further disrupt execution, while the loss of skilled professionals—especially in R&amp;D and financial management—limits organizational capacity. External constraints such as sanctions and import dependencies amplify these failures, resulting in delays, higher costs, supply-chain disruptions, and reduced competitiveness.

Overall, operational risk reflects the gap between strategic intent and execution capability, posing significant threats to operational continuity and pharmaceutical security.

### 4.5. Political Risk

Political risk was identified as a contextual factor affecting Iran’s pharmaceutical sector. Participants reported the influence of external and domestic political conditions on financing, pricing, and supply processes.

A central driver was the impact of international sanctions, which, despite formal exemptions for medicines, severely restrict banking channels and payment transfers. As one senior executive noted:

“Sanctions have practically paralyzed the import of medicines and raw materials. Even when suppliers agree, we cannot transfer payments.” (P23, Pharmaceutical Importers Association)

Domestic political volatility further amplifies uncertainty. Frequent regulatory shifts, abrupt pricing directives, and inconsistent executive orders undermine firms’ ability to plan and forecast. One financial director explained:

“We plan under one regulation today, and tomorrow a new directive cancels everything.” (P19, Parliament Research Center – Pharmaceutical Policy)

Some participants referred to issues related to resource allocation, pricing decisions, and regulatory processes. These conditions were described as affecting access to financial resources, partnerships, and supply-chain operations. Participants consistently described these influences as extending across multiple actors within the pharmaceutical system, including manufacturers, distributors, insurers, and regulatory bodies, indicating that political risk operates as a system-level exposure rather than a firm-specific challenge. Political risk was organized across four analytical dimensions: drivers and policy environment, transmission channels to firms, consequences, and control responses.

Although substantial agreement existed across stakeholder groups regarding the major financial risks affecting Iran’s pharmaceutical system, some participants expressed differing views regarding the necessity and justification of certain regulatory and policy interventions under conditions of economic instability and sanctions. These divergent perspectives were considered during the analytical process and contributed to a more comprehensive interpretation of the findings.

Beyond these differences in perspective, participants consistently emphasized that financial risks rarely occurred in isolation and were often experienced as interconnected and mutually reinforcing phenomena. Liquidity risk emerged as a central mechanism linking different categories of financial exposure. Delays in insurance reimbursements and restricted access to working capital frequently generated liquidity constraints, which increased firms’ reliance on short-term borrowing and consequently heightened exposure to interest-rate and default risks. Exchange-rate volatility further intensified these pressures by increasing the cost of imported inputs and weakening firms’ repayment capacity.

Participants also described several reinforcing feedback mechanisms. For example, liquidity shortages often led to delayed payments to suppliers and creditors, which weakened firms’ credit positions and reduced access to financing. Reduced financing capacity, in turn, further aggravated liquidity pressures. Similarly, exchange-rate instability increased production costs and financial uncertainty, contributing to liquidity constraints and greater dependence on external financing. These findings suggest that financial risks operate as a system of interconnected exposures rather than as separate categories, with effects frequently propagating across organizational and institutional boundaries.

## 5. Discussion

This study provides a cross-sectoral perspective on financial risk in Iran’s pharmaceutical system and demonstrates that these risks are interconnected rather than isolated. The findings indicate that regulatory constraints, macro-financial volatility, governance fragmentation, and weaknesses in public financial management jointly shape financial vulnerability across the sector. Consistent with international evidence (6, 7, 12-15, 17, 18), the results suggest that when pricing mechanisms lack responsiveness, reimbursement cycles are prolonged, and production systems depend on imported inputs, financial pressures become structurally embedded. In such contexts, which are common in many middle-income pharmaceutical markets, liquidity constraints, cash-flow mismatches, and exposure to political and macroeconomic instability affect operational continuity and resilience (1, 22, 24-27). A key contribution of this study is demonstrating that financial risks do not operate independently but instead form an interdependent risk architecture in which shocks in one domain, such as exchange-rate volatility or pricing distortions, propagate across liquidity, credit, and operational channels. This interconnected pattern highlights the limitations of firm-level approaches to risk management and underscores the importance of integrated, cross-sectoral governance frameworks (13, 14).

### 5.1. Liquidity Risk

The findings suggest that liquidity risk in Iran’s pharmaceutical sector extends beyond firm-level financial management and reflects broader structural imbalances in the health financing system. Delayed reimbursements from insurers, foreign exchange (FX)-denominated procurement, and inventory-related practices are consistent with mechanisms documented in other constrained pharmaceutical markets (6, 12, 27). However, this study highlights a deeper structural liquidity gap arising from the interaction of reimbursement delays, pricing rigidities, and currency misalignment. These factors reinforce cash-flow constraints and contribute to margin compression, consistent with international evidence on inventory-driven liquidity pressures (15, 22). The findings also align with prior research linking liquidity shortages to supply instability and operational disruption (17). Importantly, the results indicate that firm-level coping strategies, such as factoring or supplier credit, provide only partial mitigation. In line with integrated financial-governance frameworks (8, 14), liquidity risk in this context is better understood as a system-level structural exposure than as an isolated managerial issue.

### 5.2. Market Risks

Market-related risks in Iran’s pharmaceutical sector are shaped by the interaction of regulatory constraints and macro-financial conditions, which together create structurally embedded financial pressures. Although existing studies have highlighted the role of pricing regulation and market volatility in pharmaceutical industries (25, 27), the findings of this study indicate that, in Iran, these risks operate in an interconnected manner and reinforce one another rather than acting independently. A central finding concerns the role of pricing mechanisms as a key transmission channel of financial risk. Consistent with prior evidence, rigid and non-transparent pricing systems create misalignment between regulated prices and actual production costs. However, the results of this study show that these distortions extend beyond margin compression and trigger a broader set of firm-level responses, including stock-keeping unit reduction, quality adjustments, cross-subsidization through over-the-counter products, and tightening of distributor credit (27). This study advances the literature by demonstrating how pricing risk acts as a central transmission channel: it intensifies liquidity stress, magnifies exchange-rate exposure, and suppresses investment capacity. Strengthening pricing governance with transparent methodologies and predictable revision cycles is therefore essential for restoring financial viability (14, 27). The findings show that interest-rate risk functions as a significant amplifier of liquidity strain and investment decline. Borrowing costs consistently exceeded operating margins, while unpredictable policy-driven rate shifts increased real repayment burdens, consistent with evidence from capital-intensive regulated sectors (28). Delayed payments from insurers (6 - 12 months) forced firms to rely on costly short-term credit or accelerate receivable discounting, tightening rollover risks and elevating default probability, in line with weaknesses in public financial management reported internationally (6). Strategically, firms postponed innovation, halted expansion projects, and shifted toward short-termism, reinforcing global concerns that sustained financial pressure diminishes competitiveness (28, 29). These findings underscore the need for sector-level reforms to stabilize financing channels and improve payment discipline (30, 31). Exchange-rate volatility emerged as one of the most pervasive structural risks. Heavy reliance on imported active ingredients, fragmented FX allocation policies, and multi-rate currency regimes prevented cost forecasting, widened the gap between real expenditures and administered prices, and deepened liquidity risks, consistent with the global literature (32). The study identifies how FX shocks propagate across subsystems, including rising working-capital requirements, erosion of operating margins, increased safety-stock needs, and declining trust from foreign suppliers. These cascading effects mirror international evidence linking currency risk to innovation decline and medicine shortages. In this context, FX exposure is not merely a market risk but a systemic amplifier of financial fragility. Unlike mature pharmaceutical markets that rely on structured credit infrastructures and clearly defined market-maker roles, the Iranian system lacks a legal, regulatory, or operational framework for such functions. This study demonstrates that the resulting institutional vacuum creates a structurally “orphaned” liquidity-stabilization function, leaving suppliers, manufacturers, and distributors exposed to cascading payment delays. Without mechanisms such as clearing platforms, enforceable settlement timelines, or structured discounting systems, liquidity traps intensify, echoing evidence on weak financial-governance environments in health systems (6). This finding highlights a previously undocumented governance gap that contributes to systemic fragility. Competitive risk was driven not by market rivalry but by structural asymmetries, including preferential access to FX, licensing, or distribution opportunities for certain state-affiliated firms. Excessive fragmentation among small generic producers, destructive discounting, and opaque commercial practices further distorted incentives, echoing patterns seen in rent-seeking or price-based markets (33, 34). These dynamics suppressed innovation investment and heightened supply volatility. Mitigation requires reinforcing competitive neutrality and establishing transparent regulatory oversight (30).

### 5.3. Credit Risks

Credit-related risks in Iran’s pharmaceutical sector extend beyond conventional debt exposure and reflect deeper structural deficiencies in financial governance and market infrastructure. Although prior studies have linked default risk in pharmaceutical firms to revenue volatility and regulatory constraints (35), the findings suggest that default vulnerability is intensified by the simultaneous presence of short-term debt concentration, foreign-currency liabilities, and unstable cash-flow cycles. In particular, foreign-currency debt can increase firms’ exposure to repayment difficulties during exchange-rate shocks in emerging markets (36). A key insight emerging from this study is the role of informal credit-rating dynamics in amplifying financial vulnerability. In the absence of standardized and transparent credit-reporting systems, lenders rely on incomplete or imperfect information when assessing firms’ creditworthiness, increasing information asymmetry and uncertainty in lending decisions (37). Unlike formal rating downgrades observed in developed financial systems, these informal assessments may prompt lenders to adopt more conservative credit policies, such as stricter collateral requirements, reduced credit availability, or less favorable lending terms. This mechanism creates a self-reinforcing financial cycle in which perceived risk leads to restricted liquidity, which in turn further increases actual default risk, an interaction less explicitly addressed in the existing literature (38). In addition, the study highlights how counterparty risk and credit risk are tightly interconnected within the pharmaceutical supply chain. Payment delays by insurers and distributors not only generate liquidity pressure but also propagate upstream, weakening firms’ repayment capacity and increasing their exposure to default. This finding extends prior research on supply-chain financial contagion (14) by demonstrating how institutional payment inefficiencies in health systems can directly translate into credit instability at the firm level. The findings therefore suggest that firm-level responses, such as short-term refinancing or greater reliance on collateral, may be insufficient when used in isolation. Mitigating credit risk may instead require system-level interventions, including more transparent credit-reporting frameworks, improved financial disclosure practices, and more effective receivables and settlement processes. Improved credit information and receivables management may reduce information asymmetry and support more stable credit relationships (38, 39).

### 5.4. Political Risk

Political risk in Iran’s pharmaceutical sector operates not merely as an external constraint but as a system-level force that shapes and amplifies other financial risks. Prior studies have documented the impact of sanctions and policy instability on healthcare systems (40, 41). Domestically, frequent policy changes, evolving pharmaceutical pricing policies, and weaknesses in public financial management created uncertainty that undermined long-term planning, consistent with challenges reported in the health-governance literature (41, 42). The findings of this study suggest that, in Iran, political risk functions as a central coordinating driver of financial vulnerability rather than as an isolated contextual factor.

A key insight is that political interventions, particularly in pricing, foreign exchange allocation, and regulatory enforcement, create persistent misalignments between cost structures and revenue mechanisms. Unlike market-driven environments, in which firms can adjust to external shocks, the combination of administered pricing and politically driven decision-making restricts firms’ ability to respond adaptively. This results in a structurally constrained system in which financial pressures accumulate rather than dissipate.

Moreover, the study highlights how political risk propagates across financial subsystems. Sanction-induced restrictions on banking and international payments increase reliance on intermediaries, elevate transaction costs, and intensify exchange-rate exposure. Simultaneously, domestic policy volatility, manifested through abrupt regulatory changes and discretionary allocation of resources, undermines predictability in both financing and production planning. These dynamics create a cascade effect in which political uncertainty translates into liquidity shortages, credit tightening, and supply-chain disruptions. Another important contribution of this study is demonstrating that political risk in this context is not only exogenous but also institutionally embedded. The lack of regulatory consistency, transparency, and accountability transforms routine policy actions into sources of financial instability. This finding suggests that governance structures themselves can act as endogenous generators of risk within pharmaceutical systems. Overall, the findings indicate that firm-level adaptation strategies, such as supplier diversification or inventory buffering, are inherently limited under conditions of sustained political instability. Effective mitigation therefore requires institutional-level reforms, including greater regulatory predictability, depoliticization of pricing and resource allocation, and the development of more resilient financial and trade infrastructures.

### 5.5. Operational Risk

Operational risk in Iran’s pharmaceutical sector extends beyond routine execution failures and reflects structural inefficiencies embedded in the institutional and administrative environment. Although prior research has associated operational risk with process fragmentation and capacity limitations in constrained markets (13, 43), the findings of this study indicate that, in Iran, operational risk is intensified by the interaction of regulatory complexity, administrative inconsistency, and weak system integration.

A key insight is that the coexistence of multiple regulatory bodies and overlapping administrative procedures creates coordination failures across critical operational processes, particularly in procurement, customs clearance, and financial transactions. These inefficiencies not only increase transaction costs but also introduce delays and uncertainty that disrupt production planning. Unlike more integrated systems, in which operational risks can be managed through internal optimization, the Iranian pharmaceutical sector faces externally imposed operational constraints that limit firms’ ability to improve efficiency independently.

The study further demonstrates that operational risk is closely linked to supply-chain dependency and import reliance. Given the sector’s heavy dependence on imported raw materials, even minor administrative or logistical disruptions, such as delays in customs clearance or regulatory approvals, can rapidly escalate into production interruptions and medicine shortages. This finding extends the existing literature (14, 44) by showing how operational risk in import-dependent pharmaceutical systems becomes highly sensitive to institutional bottlenecks rather than to purely operational inefficiencies.

Another important contribution is the identification of digital and managerial capability gaps as amplifiers of operational risk. Limited digital integration across supply-chain and financial processes reduces transparency, weakens coordination, and constrains real-time decision-making. At the same time, shortages of specialized human capital and project-management capabilities hinder firms’ ability to respond effectively to disruptions. Overall, the findings suggest that mitigating operational risk requires a dual-level approach. While firm-level interventions, such as process optimization, digitalization, and capacity building, are necessary, they are insufficient without broader institutional reforms aimed at streamlining regulatory processes, improving interagency coordination, and reducing administrative complexity. This underscores the need to reconceptualize operational risk as a system-level constraint rather than a purely internal organizational issue.

### 5.6. Limitations

This qualitative analysis was designed to capture expert perspectives rather than quantify financial risks. Although participants were drawn from diverse institutional domains, the findings may not encompass all viewpoints within the pharmaceutical system. The study also focused primarily on stakeholders’ experiential insights without parallel integration of financial datasets. These limitations do not detract from the analytical contribution of the work, but they identify areas in which future research could complement the present findings using quantitative or mixed-methods approaches.

### 5.7. Conclusions

This study provides the first cross-sectoral examination of financial risks in Iran’s pharmaceutical system, demonstrating that vulnerabilities arise not from discrete operational failures but from the interaction of macroeconomic instability, regulatory fragmentation, and institutional constraints. Liquidity pressures, volatile pricing and exchange-rate regimes, weak credit governance, implementation deficits, and pervasive political risk collectively form a systemic risk architecture that undermines financial resilience and threatens medicine security. In this interdependent structure, pricing distortions, FX misalignment, and liquidity gaps cascade into credit, market, and operational failures, revealing governance fragmentation as a central structural determinant of systemic vulnerability.

Strengthening financial stability in the pharmaceutical sector therefore requires an integrated policy response, one that aligns public financial management with predictable reimbursement flows, enhances transparency in pricing and credit allocation, institutionalizes market-stabilization mechanisms, and reinforces regulatory coherence insulated from short-term political pressures. In particular, the development of transparent and evidence-based pricing systems, the establishment of national market-making and clearing infrastructure, and the strengthening of payment discipline emerge as critical priorities. At the organizational level, firms must complement these reforms through improved demand forecasting, digital integration of financial processes, diversification of financing sources, and more systematic credit-risk management.

Given the sector’s strategic importance for national health security, addressing these systemic vulnerabilities is essential not only for the financial sustainability of firms but also for ensuring equitable and uninterrupted access to essential medicines.

ijpr-25-1-170273-s001.pdf

## Data Availability

The data are available from the corresponding author on request.
