# Supplementary material for: Mapping the Financial Risk of Iran’s Pharmaceutical System: A Cross-sectoral Qualitative Study
Source: Iran J Pharm Res. 2026 Aug 19;25(1):e170273. doi: 10.5812/ijpr-170273 (PMC13681383; doi:10.5812/ijpr-170273)
Supplement: Supplementary file 1 [file ijpr-25-1-170273-s001.pdf]

Appendix1

COREQ Checklist

Consolidated Criteria for Reporting Qualitative Research (COREQ)
Fill in the "Reported on Page No." column based on your manuscript.

Topic	Item No.	Guide Questions/Description	Reported on Page No.
Interviewer/facilitator	1	Which author/s conducted the interview or focus group?	The interviews were conducted by P.Z
Credentials	2	What were the researcher’s credentials? e.g., PhD, MD	PhD candidate
Occupation	3	What was their occupation at the time of the study?	At the time of the study, the researcher was a PhD student at the Faculty of Pharmacy.
Gender	4	Was the researcher male or female?	Female
Experience and training	5	What experience or training did the researcher have?	The researcher had prior academic training and practical experience in qualitative research methods, thematic analysis, and conducting semi-structured interviews.
Relationship established	6	Was a relationship established prior to study commencement?	No prior personal relationship was established with participants before the study.
Participant knowledge of interviewer	7	What did participants know about the researcher?	Participants were informed that the interviewer was a PhD student at the Faculty of Pharmacy conducting an academic research project. They were also informed about the study objectives, interview procedures, and confidentiality of the data before participation.

Interviewer characteristics	8	Bias, assumptions, reasons and interests in the research topic	The researcher acknowledged that her academic and professional background as a PhD student at the Faculty of Pharmacy could potentially influence data interpretation. To minimize potential bias, reflexive attention was maintained throughout the research process. Interview transcripts, codes, and emerging themes were reviewed repeatedly and discussed collaboratively with other members of the research team. Differences in interpretation were resolved through discussion and consensus to enhance reflexivity, analytical rigor, credibility, and trustworthiness of the findings.
Methodological orientation and theory	9	What methodological orientation underpinned the study?	The study was conducted using a qualitative thematic analysis approach.
Sampling	10	How were participants selected?	Participants were selected through purposive and snowball sampling based on their relevant experience and expertise, and data collection continued until thematic saturation was achieved.
Method of approach	11	How were participants approached?	Participants were approached via telephone calls and text messages and were invited to participate voluntarily in the study. All interviews were conducted face-to-face.
Sample size	12	How many participants were in the study?	A total of 35 participants were recruited, and data collection continued until thematic saturation was achieved.
Non-participation	13	How many refused or dropped out? Reasons?	No participants refused to participate or dropped out of the study.
Setting of data collection	14	Where was the data collected?	Interviews were conducted face-to-face at participants' workplaces or offices.

Presence of non-participants	15	Was anyone else present besides participants and researchers?	No non-participants were present during the interviews.
Description of sample	16	Important characteristics of the sample	Participants were experts and stakeholders from policy-making, management, academia, industry, commerce, community pharmacy, regulatory organizations, insurance, and clinical sectors. Eligible participants had at least five years of relevant professional experience and were recognized nationally for their expertise in their respective fields.
Interview guide	17	Were interview questions/prompts provided or pilot tested?	A semi-structured interview guide was developed based on the study objectives and expert opinions. The interview guide was pilot tested before data collection, and the interview questions were subsequently refined by the research team.
Repeat interviews	18	Were repeat interviews carried out?	No repeat interviews were conducted.
Audio/visual recording	19	Was recording used to collect the data?	All interviews were audio-recorded with participants' consent.
Field notes	20	Were field notes made?	All interviews were audio-recorded with participants' consent and were transcribed verbatim for analysis. Field notes were also taken during and immediately after the interviews.
Duration	21	What was the duration of interviews/focus groups?	The average duration of the interviews was approximately 70 minutes.
Data saturation	22	Was data saturation discussed?	Data collection continued until thematic saturation was achieved and no new themes or concepts emerged from the interviews.

Transcripts returned	23	Were transcripts returned to participants?	Interview transcripts were returned to participants for comments, review, and confirmation of accuracy.
Number of data coders	24	How many coders coded the data?	Three researchers were involved in the coding process, and coding decisions were reviewed collaboratively throughout the analysis.
Description of coding tree	25	Was the coding tree described?	Initial codes were organized into categories and themes through an iterative analytical process. The coding structure and theme development were reviewed and refined collaboratively by the research team.
Derivation of themes	26	Were themes derived from the data?	An inductive thematic analysis approach was used, in which themes were derived from the interview data through iterative coding and constant comparison.
Software	27	What software was used?	The interview data were managed and analyzed using MAXQDA(2022) software.
Participant checking	28	Did participants provide feedback on findings?	Participants were invited to review and provide feedback on the findings to enhance the credibility of the analysis.
Quotations presented	29	Were quotations presented to illustrate findings?	Direct quotations from participants were used to illustrate and support the major themes identified in the analysis.
Data and findings consistent	30	Was there consistency between data and findings?	Consistency was maintained between the interview data, participant quotations, and the reported findings throughout the analysis.
Clarity of major themes	31	Were major themes clearly presented?	

Clarity of minor themes	32	Were minor themes/diverse cases discussed?	

Appendix2

Semi-Structured Interview on Financial Risk Management Strategies for Iran's Pharmaceutical System from a Cross-Sectoral Perspective

Semi-Structured Interview Guide

This interview aims to explore financial risks affecting Iran's pharmaceutical system from a cross-sectoral governance perspective. The study seeks to understand the experiences and perspectives of key stakeholders involved in policy-making, regulation, financing, and implementation within the pharmaceutical sector.

The interviews focus on identifying major financial risks, their underlying causes, their effects on the pharmaceutical supply chain, and potential strategies for improving financial sustainability and system resilience.

Participation in this interview is entirely voluntary. Participants may decline to answer any question or withdraw from the interview at any stage without any consequences. All information and responses provided during the interview will be treated confidentially and used solely for research purposes.

1. From your perspective, what are the most important financial challenges currently affecting Iran's pharmaceutical system?
2. Which financial risks have the greatest impact on the performance and sustainability of different stakeholders within the pharmaceutical system?
3. What factors contribute to the emergence of financial risks in the pharmaceutical sector?
4. How do financial risks affect relationships and interactions among different stakeholders, including regulators, insurers, financial institutions, manufacturers, distributors, healthcare providers, and patients?
5. In your experience, how do financial problems originating in one part of the pharmaceutical system spread to other sectors or institutions?
6. Which policies, governance arrangements, or institutional practices contribute to financial stability or instability across the pharmaceutical system?
7. How do broader economic conditions, regulatory decisions, and financing mechanisms influence financial risk within the pharmaceutical sector?
8. To what extent do different financial risks interact with and reinforce one another across organizational and institutional boundaries?
9. What are the main consequences of these financial risks for medicine availability, pharmaceutical firms, healthcare organizations, and patients?
10. What mechanisms are currently used to manage financial risks, and how effective are they?
11. What policy, managerial, or structural reforms would strengthen financial resilience across the pharmaceutical system?
12. Are there differences in how various stakeholder groups perceive financial risks and their consequences? If so, how?

Appendix 3

	Basic Code	Theme	Theme	Categories
In many pharmaceutical companies, excessive focus on short-term decisions and increased sales volume has led to lower priorities for liquidity management, resulting in disruption of the company's real cash flow.	Structural weakness in liquidity management	Structural and managerial weakness in pharmaceutical firms	Drivers / Policy Environment	Liquidity Risk
	Frequent change of managers and unstable decisions			
	Companies are focusing on selling more than just collecting demands.			
In the pharmaceutical chain, liquidity is mainly locked at the beginning of the process, especially in plasma companies, where cash payments are required, but the collection of claims from insurance companies is done with a delay of several months.	The necessity of cash settlement in plasma and raw materials companies	Cash pressures in the supply chain		
	Cash outflow from companies due to high dividends			

	Prolongation of the Order-to-Cash (O2C) Cycle in the Pharmaceutical Value Chain			
Bank financing is almost out of reach, rates are high, and banks don't have cash. Companies only settle old debt with new loans.	High interest rates and restrictions on access to bank loans	Limitations of Financing and the Banking System		
	Banks' inability to provide new credit			
	High cost of real interest compared to industry profit margin			
More than half of the sales of pharmaceutical companies are made to the public sector, but the funds from these sales are paid with a delay of several months. These continuous delays have put additional pressure on the liquidity of the companies and have brought the situation to the stage of crisis.	The size and high share of the government in the drug market	Heavy dependence on the government structure and delays in payments		
	Delay in payment of claims by insurance companies and government hospitals			
	Fragmentation of payment institutions and lack of a single settlement system			

The lack of accurate data on the timing and amount of payments has made it impossible to plan and manage cash flow effectively. This lack of transparency has significantly increased the liquidity risk of companies.	Multiplicity of actors in the chain (broadcasting, pharmacy, insurance, Ministry of Health)	Weakness of DrugSupply Chain Data		
	Weakness in Monitoring and Forecasting Operating Cash Flow			
	Lack of transparent financial data and a coherent information structure			
	Lack of data in pharmacies			
While the inflation rate in most sectors of the economy is around 50 percent, the increase in drug prices has been less than 20 percent. This discrepancy has caused the cash flow of pharmaceutical companies to be continuously weakened and their real profit margins have decreased.	High real interest rates compared to pharmaceutical industry returns	Fiscal Imbalance and Inflationary Pressure		
	Drug prices lag behind the country's general inflation			

	Widening the gap between the cost of production and the incoming cash flow			
The time interval between order placement and receipt of sales in the pharmaceutical industry usually takes several months. This long cash cycle causes a significant portion of companies' financial resources to be locked up along the supply and distribution chain.	Multiple intermediaries and stakeholders in the drug supply chain	Longevity of the supply chain	Transmission channels to the firm	
	Increase supply time from order to final sale			
	Blocking of liquidity in the supply and distribution route			
Because of the fear of shortages, we store more medicines, but this causes the working capital to be paralyzed.	[1] High DIO Shelf Life	Increasing the Inventory Turnover and Cash Lock-up		
	Maintain excess inventory to avoid drug shortages			
	Locking up capital in the warehouse and reducing free cash			

[1] Days Inventory Outstanding

The weakness in the demand forecasting system in the pharmaceutical industry has caused the balance between supply and market needs to not be properly established, so that in some cases, we are faced with a shortage of best-selling drugs and in other cases, we are faced with the accumulation of low-circulation drugs in warehouses.	Lack of an accurate and data-driven forecasting system	Weaknesses in inventory management and forecasting		
	Error in Forecasting Actual Demand (High MAPE[2], High [3]Backorder)			
	Lack of coordination between logistics, distribution and sales in manufacturing companies			
In order to maintain or increase market share, drug distribution companies in many cases place more orders than the actual need. This behavior causes the accumulation of inventory at the producer level and ultimately the cash resources of the companies are locked.	Channel Stuffing	Unrealistic Behaviors in Supply and Demand		

[2] Mean Absolute Percentage Error
[3] When a customer places an order but the company doesn't have the goods at that moment and needs to deliver later, that order enters a "backorder."

	Accumulation of inventory at the bottom of the chain (Bullwhip Effect)			
	Creating induced demand to maintain market share by manufacturing companies			
There is no transparency in some purchases, some suppliers choose from certain routes, which in itself creates financial pressure and corruption.	Purchases outside the formal and non-transparent procedure	Corruption and inefficiency in procurement		
	Conflicts of Interest in Pharmacy Purchasing Decisions			
	Supply of low-quality raw materials to save money			
Exchange rate fluctuations cause pharmaceutical companies to purchase raw materials quickly and extensively to prevent further price increases. Although this precautionary behavior is done with the aim of timely supply, in practice, it causes excessive accumulation of inventory and locking up liquidity in warehouses.	Aggressive or Speculative Orders in a Period of Currency Volatility	Price volatility and speculative behavior		

	A Sudden Change in Pricing Policies			
	Increase in the cost of recurring orders due to price instability			
The company has to pay the supplier in advance, but the money comes from the insurance six months later, which means that liquidity is always negative.	Cash payment to the supplier in exchange for delayed collection from the insurances	Cash Flow Mismatch		
	Lengthy period of receipt from distributors or pharmacies (high DSO[4])			
The cost of transportation and warehousing has now become a crisis in itself; practically part of the company's cash is burned in these circles.	Increasing the costs of transportation, distribution, and warehousing in inflationary conditions	Logistics and warehousing costs		
	Cash lock-up on intermediary fees and services			
	Increased warehouse costs due to the storage of sensitive or special drugs			

[4] Days Sales Outstanding(Claims Receipt Period)

The delay in insurance and the return of the paycheck make it impossible for us to plan for liquidity; every month a part of the uncollected claims remains	Inconsistencies in invoices and insurance documents	Contradictions in documents and delays in settlement		
	Refund of Distribution or Pharmacy Checks			
	Informal Payments and Default Risk			
When liquidity is locked, the first place to be hit is production; neither raw materials are imported, nor are broadcasters willing to buy goods without money	Stopping production lines due to lack of cash for raw materials	Disruption in the production and distribution of medicines	Consequences	
	Reduction of Pharmaceutical Raw Materials (APIs) and Packaging Items			
	Limitation of distribution and delivery capacity to pharmacies due to lack of cash			
If there is no cash, everything is locked; workers' salaries, bank installments, and taxes are not paid on time.	Inability to pay salaries and benefits on time	Lack of cash (Cash Crunch) and inability to fulfill obligations		
	Delay in paying bank or tax debts			

	Inability to pay domestic and foreign suppliers			
	Increasing effective interest rates and short-term financing costs	Increasing financial costs and decreasing profitability		
	Growth in Warehousing and Logistics Costs Caused by Delayed Sales			
	Decrease in net profit margin due to cash pressure			
Development projects have almost stopped; when we don't have money for salaries, the thought of development or R&D is meaningless .	Postponement or Cancellation of Development Projects (CAPEX[5])	Impact on Investment and Development Planning		
	Reducing Research and Development (R&D) Costs			
	Falling behind competitors in upgrading production technology			
Because of the delay in payment, the foreign supplier asks us for a down payment, which means that the financial trust has been lost .	Reducing the company's credit rating with banks and suppliers	Reducing business credibility and weakening relations with stakeholders		
	Loss of business credibility with broadcasters and foreign partners			

[5] CAPEX stands for Capital Expenditures, which is called Capital Expenditures or Investment Expenditures.

	Increasing demand for collateral and advance payments in contracts			
When production stops or cash doesn't circulate, pharmacies report shortages, and prices rise in the gray market.	Reduction in the supply of medicines and the occurrence of a temporary shortage in the market	Market instability and disruption in the value chain		
	Pressure on downstream loops (dispensers and pharmacies)			
	Increasing price fluctuations and speculative behaviors			
Currently, the granting of credit to pharmacies is not done indefinitely; a specific credit limit has been set for each pharmacy and sales to high-risk hospitals are done only by receiving a down payment.	Revision of Customers' Credit Policy	Managing Credits and Customer Relationships	Controls & Responses	
	Defining Credit Ceilings for High-Risk Buyers			
	Continuous monitoring of customers' settlement status and payment history			

	Reducing the share of sales to the public sector or indebted hospitals<br><br>and restricting or stopping cooperation with high-risk entities	Controlling Exposure to Government Buyers		
	Condition of prepayment for sale to certain centers			
We are now working with the Innovation Fund because the bank does not give loans; these resources are cheaper and have a longer breathing period.	Using development and support resources (Innovation Fund, National Development Fund) for the pharmaceutical industry	Structural and Development Financing		
	Receiving long-term facilities for domestic production projects			
Continued dependence on short-term banking facilities is not sustainable; we must gradually move to structured and capital market instruments for financing, including sukuk issuance, corporate debt securities, structured factoring, and receivables securitization, which are designed based on assets and cash flow.	Replacing Short-Term Bank Financing with Structured Instruments			

To maintain production, we rented our equipment and used the cash for working capital, and then we rented the same equipment again.	Using the Sale & Leaseback Model to Release Cash[6]	Asset-based financing		
	Re-leasing of productive assets to maintain operation			
	Transferring part of fixed assets to short-term cash flow			
We issued debt securities to cover the gap between the purchase of materials and the receipt from insurance; without it, liquidity would not have been possible.	Issuance of debt securities to compensate for cash shortage	Financing from the capital market		
	Adjusting due dates according to the production and sales cycle			
In order to make the right financing decisions, it is necessary to compare the effective interest rate (EIR) of each instrument with the bank interest rate, because sometimes the actual cost of factoring, sukuk, or short-term lines of credit is lower than the nominal bank loan rate.	Comparison of the cost of financing (EIR)[7] with the bank interest rate			

[6] The Sale & Leaseback model refers to a financial instrument to release liquidity from fixed assets.
[7] Effective Interest Rate

Shareholders were forced to inject cash, because without it, it would not even be possible to pay salaries	Increase in capital from cash or accumulated profits	Strengthening domestic capital and shareholders		
	Improving the ratio of financial leverage and resilience to liquidity shocks			
We agreed with the foreign supplier to extend the payment deadline to 90 days, because liquidity was really limited.	Increased payment deadlines (DPO)[8] from domestic and foreign suppliers	Use of suppliers' credit		
	Negotiating an extension of the settlement period in exchange for maintaining long-term cooperation			
We told the broadcasters that if they settle early, they will get a percentage discount, which led to the cash coming back sooner.	Dynamic Discounting	Cash incentives for quick settlement		
	Fast cash clearance to motivate customers			
For certain medicines, we agreed with the insurance to take part of the amount before production to make it easier to provide currency.	Prepayment Contracts for Bulk Orders	Pre-receipt and deposits of customers		

[8] Days Payable Outstanding

	Receiving deposits from insurance companies or purchasing institutions to provide pre-production cash			
In the current situation, the company's claims from the insurers are transferred to the factoring companies to speed up the process of converting them into liquidity, although this method requires the payment of a significant fee or fee.	Sale of insurance claims and distribution to financial institutions	Factoring and Discounting of Claims		
	Reducing DSO [9]through discounting at a lower effective rate of bank interest			
In order to manage the critical situation and prevent the suspension of essential payments, a short-term credit line has been received from the bank to provide immediate liquidity in case of emergency.	Using Short-Term Credit Lines (Overdraft)	Credit lines and revolving loans		

[9] The term DSO stands for Days of Sales Outstanding, which is called the period of collection of receivables for the duration of the collection of sales.

Optimal management of credit line utilization rates and effective interest rate control is vital, because if short-term resources are used too much or at high rates, the company's actual financial cost will be much higher than the nominal bank loan rate.	Effective utilization rate[10] and cost management			

**Proposition**	Basic Code	Theme	Theme	Categories
Pharmaceutical companies abandon the production of some products due to the low profit margins, which leads to a decrease in diversity and a therapeutic gap in the market.	The Cost of Opportunity for Companies to Produce a Product	Financial and cost pressure	Drivers and the political environment	Price Risk
The increase in the interest rate of bonds compared to the profit margin of the drug makes it difficult for manufacturers to finance and reduces the attractiveness of investment in the	Bond interest rates higher than drug profit margins			

[10] Utilization Rate: The ratio of the actual use of credit lines or facilities to their approved ceiling. It means how much of the resources available to the company has actually consumed.

pharmaceutical industry.				
The inability of the pricing system to cover the real financial and operational costs causes double pressure on companies and reduces their competitiveness.	The Cost of Living in the Philippines: Lack of Transparency			
The lack of sufficient motivation to develop the product portfolio in the shadow of restrictive policies hinders the supply of new drugs and increases the choice power of patients.	Failure to complete the product portfolio	Shortages and supply constraints		
Drugs that are financially profitable are not prioritized for production, and this leads to chronic shortages in the country's pharmaceutical market.	Shortages in unprofitable drugs			
The lack of a codified calendar and the multiplicity of decision-making	Lack of a price revision calendar and fragmentation of decision authorities	Institutional and regulatory inefficiencies		

centers cause the price revision to not be done in a timely manner and instability prevails in the pricing system.				
The low expert capacity and limited human resources in the pricing commission make the decision-making process inefficient and time-consuming.	Inefficiency of pricing commissions			
The stability of the old regulations and the lack of transparency of the regulations confront the pricing process with ambiguity and limit the possibility of adapting it to the current conditions.	Old or obscure regulations and laws (such as the 1955 law)			
Widespread government intervention in setting and controlling prices weakens the competitive environment and reduces the incentive to	Direct government intervention in control mechanisms	Prescriptive policies and government interventions		

innovate in pharmaceutical companies.				
Directive policies in determining drug prices prevent flexibility and innovation, and prevent companies from developing their products in accordance with real costs and market conditions.	Lack of development in order pricing			
The monopoly of some companies and the occurrence of violations in the market disrupt healthy competition and affect patients' equitable access to medicine.	Occurrence of Violation and Existence of Monopoly	Corruption and unhealthy competition		
Lack of transparency in the pricing process leads to corruption and actions such as factoring to obtain unrealistic prices.	Creating corruption in inappropriate pricing methods; factoring in pricing			
There is not enough expert staff in the pricing commission, and this makes the	Lack of specialized human resources in pricing commissions	Institutional Capacity and Human Resources		

reviews very time-consuming and sometimes superficial.				
Lack of training courses and insufficient familiarity with new methods cause experts to continue to rely on traditional and inefficient models.	Lack of specialized training and updates for pricing experts			
Most of the decisions are made in the closed circle of a few people, and a specialized view from other areas does not enter the process, which has reduced the quality of decisions.	The focus of decisions is limited to a few people and the absence of multidisciplinary teams.			
When they compare the price of medicines with countries whose economic structure and production costs are not the same as ours, it is natural that the final price is unrealistic	Using the Price Reference Method with Countries that Do Not Have Similar Economic Conditions	The Methodology of Disproportionate Pricing		
In Iran, there are official cost-effectiveness models that are included in the evaluation of drugs, but the	Applying cost-effectiveness and value-based models			

high volume of bureaucracy and complex administrative processes cause the efficiency of this tool to decrease and the path of drug entry into the market is prolonged.				
Pricing formulas include a portion for the cost of R&D, but its contribution is very small and, given the real inflation, it never meets the costs of innovation.	Ignoring R&D Costs in Pricing Formulas			
For example, when they compare a medicine with Turkey or India, they do not pay attention to the fact that insurance covers most of the cost and their distribution chain is much easier, but in Iran, pharmacies and insurance companies delay the money for months. These differences make the price not at all compatible with our reality.	Lack of attention to the difference between insurance structure and supply chain in Iran compared to the reference country			

	Disproportionality of pricing with the allocated exchange rate (NIMA / Azad)			
When the price of supplements was released, the competitive pressure was effectively transferred to the basic medicines, and some companies were forced to offer the original drugs below the actual price.	Liberalization of Health Supplements Prices and the Domino Effect on the Drug Market	Liberalization and Market Interactions		
Our pricing system is static; when the cost of production or the exchange rate changes, the price of medicine is not updated, and this causes companies to suffer losses.	Lack of a dynamic mechanism to match prices to market conditions			
As long as there is no currency crisis or a shortage of medicines, they will not change prices and everything will be reactive.	Price revisions can only be done after the pressure of currency crises or drug shortages.	Delay in revision of prices		
There is no set calendar for revision, we don't know when we should wait for the price correction.	Lack of a specific calendar for annual or periodic revisions of prices			

Applications stay in line for months, and this prolongation of the review causes companies to face liquidity and production problems.	Accumulation of price correction requests and lengthening the review process			
In an industry such as medicine, where costs rise monthly, keeping the price stable for two years means that the producer is practically on the path of structural loss; this is no longer a liquidity problem, it is a chronic economic pressure.	Continuous increase in production costs compared to fixed prices	The gap between the approved price and the cost is over.	Transmission channels to the firm	
When an essential drug is produced with a negative profit margin, that is, every time the production line is turned on, the company registers more losses. The continuation of production in such conditions is only due to social responsibility, not economic logic.	Negative profit margins and loss-making of essential items			

The price correction is completely manual; one item may be corrected for two weeks and the other item may wait two years. This unpredictability is catastrophic for us.	Lack of an intelligent mechanism for revising prices			
Cases of price increases are passed like a ball on a multi-level field, from expert to committee and from committee to council. No one has a time commitment. The result is that the files stay in line for months and are practically buried under a mountain of paper.	The long interval between the request for a price increase and the final decision	Delay in price correction and price freeze		
	Failure to revise the price in accordance with the fluctuations in cost and exchange rate			
	Stoppage of production or reduction of production due to losses caused by price freeze			
Since the price of the original drug has not been corrected, we have to compensate for its losses by selling supplements and OTC.	Increasing the sale of profitable items to cover the losses of loss-making items	Companies' Side Behaviors to Compensate for Inappropriate Price		

In order not to lose money, we had to get the raw material from a cheaper seller.	Reducing the quality of inputs or changing the supplier to reduce the cost			
Since the price of the product does not arrive, we postpone the payment to the supplier to preserve liquidity.	Increased debt and delays in payments to suppliers to manage liquidity			
Because of the changing price policies, no one knows if the drug will be on the market in a month.	Uncertainty and Supply/Demand Instability	Market volatility	Outcome	
Companies stop production until the price is corrected, and this causes a temporary shortage in the market.	Temporary Drug Shortage			
When it's unpredictable, some people hold the drug to sell when the price rises.	Hoarding and speculative behaviors			
Few companies are willing to invest in a new product in a situation where pricing is mandated.	Postponement or cancellation of investment and product development (opportunity cost)	Reduction in investment and development		

When innovation isn't reflected in pricing, companies prefer not to go for risky R&D projects.	Reduced Motivation for Research and Development			
Due to the delay in correcting prices, the liquidity of companies is locked up and they are forced to resort to bank loans at high rates.	Increase in bank debt and liquidity pressure	Finance & Credit		
When a company fails to meet its financial obligations, its credibility in the banking system is also damaged.	Weakening the credit rating and goodwill of companies			
Companies reduce the quality of raw materials to compensate for costs, and this directly affects the health of patients.	Decrease in production quality due to financial pressure	Operational and Quality		
When prices are not updated in time, the production schedule is constantly changing, and the stability of the production line is disrupted.	Disruption in production planning			

When the pricing of the drug does not match the cost of production, the manufacturer transfers the financial burden to the distribution company with stricter credit terms.	Transfer of losses from the manufacturer to the distribution companies	Transfer of Financial Burden		
When the difference between the actual cost and the duty price rises, insurers are forced to cover the additional burden, but they pay with long delays.	Transferring the cost to the insurance companies			
Much of this pressure is eventually passed on to the patient, either through a lack of medication or free purchase at higher prices.	Imposing the final cost on the patient			
To compensate for the delay in payments, the chain is forced to take out a high-rate bank loan, which creates double costs.	Chain Dependence on Bank Loans			

Foreign companies also limit cooperation when their money is paid late or low prices are imposed, and this increases the risk of supplying medicines.	Transfer of risk to foreign suppliers			
When we lowered the line waste, the same price became more bearable for us.	Production line optimization and waste reduction	Improved productivity and efficiency	Controls & Responses	
With long-term contracts, we stabilized the price of the API and reduced our cost volatility.	Consolidation of raw material purchases and long-term contracts			
We redesigned the distribution routes and the logistics cost of each unit dropped significantly.	Reconfiguration of the broadcast and transmission network			
We closed a few loss-making items so that our resources could be focused on vital and profitable items.	Portfolio agility (removing [11]loss-making SKUs)			

[11] Stock Keeping Unit

The Darouyar plan brought the price of medicines closer to the real level, but prevented direct pressure on the patient by transferring subsidies, which is an example of controlled liberalization in Iran's pharmaceutical system.	Darouyar plan as an example of conditional release with the transfer of subsidies to the targeted organization	Controlled Release		
	Phased liberalization in several stages			
	Pilot Liberalization in Low-Risk/Multi-Producer Groups			
	Conditional Release by Strengthening Insurance Coverage			
	Tiing liberalization to indicators (currency, materials, wages)			
	Staggered pricing based on production volume or number of producers			
	Greater liberalization in OTC and supplements, tighter control on Rx.			
	Using the Online Monitoring Dashboard to Monitor the Effects of Liberalization			

Rebate and Clawback make competition between companies healthier and prevent financial violations.	Rebate and [12]Clawback Transparency [13]to Prevent Unhealthy Competition			
	Cost-plus with standard cost audit	Pricing Methodology and Models		
	Dynamic Reference Pricing with PPP Modulation			
	Transparent tender with qualitative-technical indicators			
	Value-Oriented/Result-Based Contract with Insurance			
	Definition of Justice-Oriented Methodology			
	Increasing FTE [14]and specialized training of commission members	Institutional capacity		
	Independent Appeals and Appeals Mechanism with a Specified Deadline			
	Registration and Management of Conflicts of Interest of Commission Members			

[12] Hidden Discounts
[13] Recovery Mechanisms
[14] Full-Time Equivalent

	Increasing Commission Capacity (Human Resources/Software)			
	Automatic Monthly/Seasonal Calendar	Dynamic Review		
If one of the indicators crosses the threshold, the revision will be automatically enabled.	Objective triggers such as wage exchange rate fluctuations, producer index			
	Formula for Weighting Currency/Materials/Energy/Wages	Indexing		
	Moving Average Exchange Rate to Reduce Shock			
	Integrated pricing and workflow system	Digitization		
Companies and insurance are tied to official data, and everyone no longer negotiates with their own number.	Data API for Companies and Insurances			

Proposition	Basic Code	Theme	Theme	Categories

	Strictness in the ratio of loans to collateral [15]for pharmaceutical companies by the Central Bank of Iran	Macro and prudential policies	Drivers / Policy Environment	**Interest Rate Risk**
	Debt-to-income coverage limits			
	Increasing Risk Weight for Health Sector Loans			
	Prohibition or Limitation on the Granting of Working Capital Loans to Loss-Making Companies			
	Arrears of insurance payments (6–12 months)	Delays and Payment Pressures		
	Failure to pay claims on time by public hospitals			
	Transferring the financial burden to the distribution chain and pharmacies			
	Heavy fees in the issuance of corporate bonds	Capital Market Regulations and Costs		
	The requirement to have a credit rating for the issuance of bonds (in Iran, incomplete infrastructure)			

[15] Ltv

	The long process of obtaining a license from the Stock Exchange Organization for financing			
	Higher interest rates in the debt market than banks			
	Lack of an active market for instruments such as Interest Rate Swap (IRS)	Derivatives Limitation and Hedging		
	Unavailability of Cap/Floor Tools for Non-Financial Companies			
	Legal prohibition of interest rate hedging in domestic contracts			
	Mere dependence on bank loans without the possibility of risk diversification			
	Abrupt changes in the central bank's foreign exchange policy that shift interest rates	Currency Policy and Capital Control		
	Restrictions on the transfer of capital abroad for foreign exchange financing			
	High correlation between exchange rates and interest rates in Iran (simultaneous shocks)			

	Obligation to return the export currency at the order rate			
	Iran's low credit rating ceiling compared to the countries of the region	Sovereign/Country Risk		
	The country's high risk premium for any type of foreign financing			
	Restriction of Access to International Credit Lines			
	Increase in the spread of government bonds, which raises the cost of financing companies			
	Expected inflation rate above 30–40%, which puts pressure on nominal rates.	Inflation expectations and uncertainty		
	Fluctuation of reset rates in floating facilities			
	Lack of valid indexing for inflation management in contracts			
	Constant changes in monetary policy that reduce predictability			
	Liquidity crisis in commercial banks	Banking Network Health		
	Continuous increase in interbank rates			

	Banks' balance sheet imbalances that affect interest rates			
	Reducing the lending power of banks to the pharmaceutical industry			
	Setting an interest rate ceiling to control government spending	Government Mandate and Financial Interventions		
	Setting Interest Rate Floors to Attract Bank Deposits			
	Granting mandatory facilities to unproductive sectors that lock resources			
	Widespread issuance of government bonds and attracting market liquidity			
	Central Bank's Open Market Operations Policy	Monetary Policy and Base Rate		
	Increase in statutory deposit rates for banks			
	Creating an interest rate corridor that restricts access to banking resources			
	Spread growth between the base rate and the effective loan rate			
	The gap between interest rates and inflation that destroys companies' profitability	Instability and inefficiency of the interest rate structure		

	The unjustification of using shareholder resources for new investments			
	Push for bank financing as a tool to control inflation			
The increase in interest rates has caused a large part of the company's operating profits to be spent on paying interest on loans; in fact, production has become more expensive for us than financing.	Increasing the interest rate of short-term loans to cover current expenses	Cost of Working Capital	Transmission Channels	
	Increasing the need to provide liquidity from facilities instead of operational flows			
	Increasing banks' collateral demand and decreasing firms' bargaining power			

Due to high interest rates and delays in payments by insurances, we have to sell claims at a discount so that liquidity returns faster, even if we lose part of the profit.	Granting cash discounts to expedite cash returns from dispensaries and pharmacies	Pressure on sales cash flow		
	Applying commodity discounts to stimulate demand in a competitive market			
	Discounting insurance claims to compensate for long-term delays in high collection			
	Increased pressure on profit margins due to the combination of discounts and financial costs			
At current interest rates, corporate bonds are not economical, the government has absorbed resources with its own bonds, and the private company has no chance of financing.	Reducing the attractiveness of debt securities issuance due to high financing costs	Financing Instruments & Bonds		

	The lack of economic efficiency of using instruments such as sukuk or gam issued by the Securities and Exchange Organization			
	Weak credit rating infrastructure and difficulty in obtaining a bond issuance license			
	Competition of interest rates of government bonds with corporate bonds (crowding out)			
Due to fluctuations in interest rates and inflation, we are forced to store more raw materials, but this locks in the company's liquidity and raises the financial cost.	Increasing the opportunity cost of maintaining inventory and raw materials	Inventory and Reserves		
	The obligation to maintain a safety stock to deal with supply and inflation risks			
	Decrease in free liquidity due to locking up of capital in the balance			
	Decline in asset turnover and working capital productivity			

When the interest rate rises, a large part of the liquidity is spent on paying installments and interest, and the probability of default of the facility is greatly increased.	Increasing the cost of repaying bank facilities	Liquidity Pressure and Default	Consequences	
	Double pressure on cash flow due to maturity simultaneity and stagnant sales			
	Shifting Resources to Pay Interest Instead of Operating Investment			
At these interest rates, production projects no longer have economic justification; even a project that is profitable has a negative NPV.	Reduction of NPV and IRR of development projects	Reducing the Attractiveness of Investment		
	Postponement of projects to increase capacity or modernize production lines			

	Investors' reluctance to enter the pharmaceutical sector due to the high cost of financing			
	Shifting resources from productive investment to unproductive markets (real estate, gold, etc.)			
High interest rates have made our financial costs more than profit margins; sometimes even sales are made at a loss to provide liquidity.	Increased Cost of Production and Distribution (COGS)	Profitability and cost		
	Gross and operating profit margin compression			
	Reducing the company's ability to cover financial expenses from operating income			
	Transferring the financial cost to the price of the drug if possible or operating losses otherwise			

With these financial costs, companies prefer to survive rather than grow; R&D projects are practically dormant.	Reducing CAPEX and Stopping Infrastructure Projects	Stopping Long-Term Investments		
	Reduction of research and development (R&D) funding			
	Stopping the promotion of technology and production quality<br><br>Prioritizing Survival and Liquidity over Innovation and Development			
To control the financial cost, projects that have short-term returns go ahead, and for liquidity, we try to use factoring and negotiation with the supplier to reduce the interest pressure.	Prioritization of Less Rate-Sensitive Projects	Project Management & Finance	Controls/Responses	

	Factoring or discounting documents at a lower effective rate compared to bank loans			
	Negotiating to increase the period of payment to the supplier and reduce the period of collection of receivables			
	Redesign of Cash Flow and Internal Settlement between Group Companies			
	Using operating revenues and internal cash to finance	Diversification of Funding Sources		
We tried not to rely only on bank loans, for example, we used the Innovation Fund and customer advances to cover the costs of the project.	Utilizing the Innovation Fund and Specialized Low-Interest Facilities in the Health Field			
	Receiving advance payments from customers or long-term sales contracts			

	Use of collaborative financing tools (such as venture capital or project fund)			

Proposition	Basic Code	Theme	Theme	Categories
For the government, the supply of foreign exchange for medicine is not just a financial decision, it is a constant pressure on the country's foreign exchange reserves and administrative system.	Expensive supply of foreign exchange by the government	Regulatory Restrictions and Currency Allocation Costs	Drivers / Policy Environment	**Exchange Rate Risk**
	The lengthy administrative process of receiving government currency or half			
	Banking Restrictions on Transferring Currency to Foreign Suppliers			
	Obligation to obtain multiple licenses from the Central Bank and the Food and Drug Administration			

	Sharp exchange rate fluctuations in short periods	Currency volatility and multi-rate market		
	Multi-rate currency (governmental, half, free)			
	Sudden changes in foreign exchange policies (e.g., removal of preferential currency)			
	The impossibility of predicting the cost of medicine due to gradual changes in the exchange rate			
	Allocation of foreign exchange rents to certain companies	Structural Corruption Caused by the Multi-Rate Exchange System		
	Overprice in importing raw materials under the pretext of preferential exchange rate			
	Losses of healthy companies that do not have access to rentier currency			
	Transferring the Cost of Corruption to Patients and Ultimate Beneficiaries			

The company has to provide multiple times the liquidity to buy free currency, while order pricing does not allow to offset this cost.	The need for high initial liquidity to buy free currency	Liquidity pressure and price effects		
	The big difference between the real foreign exchange cost and the order pricing of the drug			
	Decrease in the purchasing power of raw materials due to the decline in the value of the rial			
	Increasing the Cost of Working Capital for Firms			
	Changes in the currency base of contracts by foreign suppliers (e.g. from euros to dollars or vice versa)	Changes to the contract and pricing	Transmission Channels	
	The difference between the time of currency allocation and the time of settling the contract and creating a price gap			
	Obligation to pay the difference in foreign exchange in long-term contracts			

When a contract is signed in euros, but we have to pay free dollars at the time of payment, the costs multiply and the insurance companies do not accept this difference.	Rejection of price adjustment by domestic institutions (such as insurance or the Ministry of Health)			
The Central Bank delays the exchange and the company is forced to buy from the free market temporarily, which means a multiplier overhead cost for the same import.	Delay in the allocation of government currency and the need for temporary purchases from the open market	Overhead & Financial Costs		
	Increase in banking costs and foreign exchange transfer fees			
	Re-incurred costs due to repayment or cancellation of foreign exchange transfers			
	Double pressure on working capital to provide foreign exchange advances			

If we buy free currency and the drug remains in stock, the cost of sleep will lock in the company's total liquidity.	Increasing the cost of inventory maintenance due to currency valuation	Warehouse Inventory and Strategic Reserves		
	Losses due to capital sleep in the inventory of raw materials or imported medicines			
When the currency is not allocated on time, the company's liquidity is locked and we have to take out short-term loans at a heavy cost.	Growth in the need for working capital to purchase imported raw materials	Pressure on liquidity and working capital	Consequences	
	The obligation to maintain a safety stock (mandatory balance to deal with currency risk)			
	Blocking of liquidity in the processes of allocating and approving the government/half currency			
	Increasing the cost of short-term financing (bank loans, step bonds, etc.)			

Currency fluctuations cause some manufacturers to stop production at all or their warehouse inventory becomes unusable due to the high cost.	Expiration date of raw material inventory due to currency fluctuations			
We import from free currency, but we have to sell at the approved price, the result is that the profitability of the company is severely strained.	Increase in the cost of production due to free currency	Cost pressure and profitability		
	Reduction and compression of profit margins			
	Sharp fluctuations in the selling price			
	Reduced ability to invest in research and development due to lack of resources			
	Increase in marketing and logistics costs due to currency instability			

Sometimes the company is forced to stop the production of vital medicines because its raw materials are no longer economical at the approved rate.	Stopping or reducing production lines due to shortages of imported raw materials	Reduction in production and supply capacity		
	Shortage of vital medicines in the market and patients' dissatisfaction			
	Increase in waste and scrapping of raw materials inventories due to sudden changes in the exchange rate			
	Migrating orders to less expensive drugs and abandoning innovative drugs			
If foreign exchange resources are transferred directly to insurance companies or we use domestic LCs, the risk of currency being blocked or lost is reduced.	Direct transfer of foreign exchange resources to insurance companies for settling claims	Currency Management Tools	Controls/Responses	

	Use secure settlement tools such as built-in LC or Escrow account			
	Credit insurance in foreign transactions in case of access to financial institutions			
When the supplier and the import route are diversified, the company is less involved in the risk of a particular source or bank.	Multi-sourcing of suppliers of raw materials for aggregate procurement to reduce the cost of foreign exchange	Diversification and hedging		
	Variety of import routes and use of different banks or exchanges to distribute risk			
	Insert Currency Adjustment Clause (FX)<br><br>Adjustment Clause)<br><br>In Contracts	Contractual and Price Mechanism		
	Changing the price revision period from annual to seasonal or monthly			
	Using currency indexing to match the contract to the real rate			

	Using foreign exchange earnings from the export of medicines or raw materials	Use of domestic foreign exchange resources		
	Allocation of foreign exchange revenues of co-group companies in holdings to drug imports			

Proposition	Basic Code	Theme	Theme	Categories
	Lack of direct government entry into market making	No government intervention in the market	Drivers/ Policy Environment	Market Risk
	Lack of instructive pricing by the government			
	The Regulatory Role of the State			
	Corruption in the supply chain	Inefficiency and structural violations		
	Increasing market fraud			
	Reduced actor competition	Weakening the competitive environment		
When market rules are manipulated, companies have no incentive to innovate and compete.	Decreased motivation of actors			
	Lack of payment calendar and late			

	penalties for institutional buyers	Weakness of payment mechanisms		
	Limitation of credit/discount instruments (step bonds, factoring, built-in LC)			
	Fragmentation of financial and insurance institutions, lack of a single clearing & settlement			
	Pooling of funds, liquidity management, and regular chain settlement	Inefficient management of financial resources		
	The Role of Logistics Companies as Financial Managers			
	Due to high inflation, market building does not make sense.	Macroeconomic instability		
	Extreme exchange rate fluctuations make market planning impossible.			
	Frequent changes in macroeconomic policies confuse the path of competition.			

	Complete Corporate Value Chain	Market Structure and Players		
	The monopoly of some quasi-state-owned enterprises eliminates healthy competition.			
	The broadcaster's dependence on several large private and public companies has led to unequal bargaining power.			
	There is a need for free supply and demand in the supply chain.	Disruption of the Supply and Demand Mechanism	Transmission channels to the firm	
	Distribution of drug rents through government quotas			
	Drug Depots by Distributors and Pharmacies to Profit from Price Increases			
	Artificial shortages in the market due to improper supply management			
	Slow transfer of monetary resources between the producer, the distributor and the bank	Current liquidity problem		

	Multi-Stage Settlements in the Distribution Chain			
	Lack of financial instruments such as factoring or step bonds			
	High Cost of Discounting Documents in Banks			
	Inconsistencies in documents and delays in approving insurance prescriptions	Problems with insurance and financial documents		
	Complex bureaucracy in the registration and handling of manuscripts			
	Partial rejection of documents due to non-compliance with the guidelines			
	Lack of Integration of Insurance Systems and the Food and Drug Administration			
	Prolongation of the period of collection of receivables	Blocking of capital in receivables		
	Increasing debt in government hospitals and medical centers			

	Locking up of funds in broadcast accounts receivable			
	Lack of a penalty mechanism for the delay of institutional buyers			
	Increase in fraud in the market due to lack of transparency	Spread of corruption and fraud	Outcome	
	Discounts and Invoices for Higher Prices			
	Companies Withdraw from the Production of Low-Profit Drugs	Lack of access		
When prices are unrealistic, manufacturers abandon low-profit drugs, and shortages are formed in the market.	Focus supply on high-profit drugs and abandon other products			
	A financial institution with a buffer feature to cover the insurance payment gap	Development of financial and credit instruments	Controls, Answers.	
	Designing Factoring Tools and Step Sheets for the Pharmaceutical Industry			
	Facilitating short-term credit lines for distribution companies			

	Discount of insurance claims			
	Establishing the commodity field of medicine as a transparent price reference	Transparency and Competition in Supply (Commodity Field)		
	Obligation to register broadcast transactions in a transparent system			
	Reducing rent quotas with open supply in the commodity field			
	Monitoring Drug Inventory to Prevent Self-Made Shortages			
	Entry of medicine into the commodity exchange for major transactions	Official and Observable Market Making (Drug Exchange)		
	Discovering a fair price based on supply and demand<br>Creating the possibility of financing through the capital market			
	Reducing intermediaries' rents with transparency in buying and selling			
	Financial intermediary between pharmacies and insurance for quick liquidity release	Reducing late payments (financial intermediation)		

	Payment to pharmacies in less than a month			
	Using financial management companies as payment intermediaries			
If there is a financial intermediary, the pharmacy does not have to wait a few months to receive its claims from the insurance.	Reducing the financial burden of pharmacies through tripartite contracts (insurance, pharmacy, financial intermediary)			
	Creating a centralized settlement center for the entire pharmaceutical chain	Integration of the payment system (centralized consolidation and settlement)		
	Connecting insurance, distribution and producer systems to a single platform			
	Simultaneous settlement of receivables across the entire chain to prevent liquidity from being stuck			
	Reducing document discrepancies and delays in verifying manuscripts with a single system			

If settlement systems become transparent, companies will no longer be able to play with nominal discounts or delays in payments.	Creating an integrated system for liquidity settlement and circulation	Improve transparency and oversight		
	Requirement of transparent financial disclosure by pharmaceutical companies			
	Strict monitoring of discounts and distribution contracts			
If a financial instrument such as factoring is available, the firm can release locked liquidity more quickly	Development of credit instruments such as step bonds, domestic LC factoring	Liquidity Management & Financial Instruments		
	Defining the Delay Penalty Mechanism for Institutional Buyers			
	Facilitating Companies' Access to Short-Term Loans with Preferential Interest			

If the government plays the role of the real regulator instead of acting, more fair competition will be formed.	Avoiding direct government entry into market making	Fair regulation of the government		
	Revision of order pricing policies			
	Defining the Healthy Competition Framework in Pharmaceutical Tenders			
When distribution is transparent and inventories are managed intelligently, we will no longer see artificial shortages of medicines.	Improve logistics processes to reduce cost and time	Improving supply chain efficiency		
	Participation of Private Distribution Companies in the Design of a Transparent Distribution System			
	Intelligent inventory management to reduce fictitious shortages			

	Supporting research and development in pharmaceutical companies	Enhancing Investment and Competitive Motivation		
	Tax incentives for investment in domestic production			
	Supportive Policies for the Production of Low-Profit and Vital Drugs			

Proposition	Basic Code	Theme	Theme	Categories
Competitive risk for firms is exacerbated when exchange rate fluctuations not only destabilize the cost and pricing structure, but also reduce strategic predictability, effectively turning a sustainable competitive advantage into an ongoing challenge.	Exchange rate fluctuations	Macro Policies and Market Regulations	Stimulus/Policy Environment	**Competitive Risk**
Imposing restrictions or bans on the import of medicines and raw materials causes the fragility of the supply chain and disrupts the competitive conditions.	Import Restrictions			
Setting a price ceiling by policy-making bodies limits the freedom of action of companies in pricing.	Order Pricing			
Government interventions in import tariffs, currency	Direct government interventions			

allocation, and price control lead to the formation of unhealthy competition in the drug market.				
The high concentration of large companies or the excessive dispersion of small players affects the intensity of competition in the drug market.	Cast concentration or dispersion	Market Structure and Conditions		
The increasing therapeutic complexity and specialization of drugs make it difficult for competitors to enter and change the competitive pattern of the market.	Level of Specialization of Treatments			
The limited size of the domestic market or fluctuations in demand intensify the competition for market share.	Market size and demand capacity			
The ability of companies to integrate the supply chain (from raw material to distribution) can create a competitive advantage and increase pressure on competitors.	Supply Chain Integration			
Preferential relationships and bargaining power of distribution companies in interaction with suppliers and pharmacies lead to unequal access and unhealthy competition in the drug market.	Distribution and Access Channels			
	Large dispersion of generic			

	pharmaceutical companies			
Reducing the level of insurance obligations and increasing the share of out-of-pocket payments intensify competition for more economical drugs.	Limitation of Insurance Coverage	Insurance Coverage System and Market Access		
The lengthy and bureaucratic process of obtaining a license to import or produce drugs delays the entry of competitors and creates unequal conditions in the market.	Complex licensing process			
The gap between basic and supplementary insurance coverage becomes critical when the actual health needs exceed the level of basic insurance liabilities, while supplementary insurance is either costly or lacks the necessary comprehensiveness, resulting in increasing financial pressure on households and a decrease in the effectiveness of the health financing system.	Gap between basic and supplementary insurance coverage			
The budget constraints of insurance in expensive health care groups create fierce competition among companies for coverage.	Budgetary constraints of insurances			
The use of exaggerated and induced advertising methods in the introduction of drugs misleads doctors	Seductive propaganda	Unethical Behaviors and Lack		

and intensifies unhealthy competition in the market.		of Transparency in Drug Marketing		
The use of non-transparent methods such as gifts, commissions, or informal payments to doctors distorts the atmosphere of competition.	Marketing Violations			
The lack of binding guidelines and efficient monitoring of promotion activities increases advertising abuses and undermines healthy competition.	Lack of a binding framework for promotion			
The inequality of access to scientific, financial, and market information between companies, doctors, and patients causes abuse by some actors and disrupts the competitive transparency of the pharmaceutical market.	Asymmetric information in the market			
The lack of transparency in the reporting of marketing and sales costs leads to the creation of rents and unhealthy competition.	Weakness of Pharmaceutical Companies' Financial Disclosure			
The opaque financial relationships between some doctors and pharmaceutical companies reduce public trust in prescriptions and undermine ethical competition.	Conflict of Interest between Doctors and Companies			
The rapid entry of competitors and the widespread supply of generics reduces the market	Decline in the market share of major companies	Destructive Mechanisms of Competition in the	Transmission Channels	

share of leading companies and intensifies price competition.		Pharmaceutical Market		
The use of methods such as forced bundling, opaque discounts, and informal agreements disrupts the structure of healthy competition and disrupts distributive justice.	Unhealthy competition in the market			
The imposition of heavy and unrealistic discounts by some companies to attract the market leads to the elimination of competitors and instability in the pharmaceutical industry.	Unrealistic and Destructive Discounts			
The strong presence of state-owned pharmaceutical companies with the benefit of supportive rents creates unfair competition for private companies.	Ownership Structure and Unequal Competition			
Changing suppliers in the drug market is costly and difficult due to the need for technical and regulatory approvals.	High Cost of Supplier Change	Barriers to Displacement and Dependence in the Drug Supply Chain		
Long-term contracts with distribution companies or manufacturers limit the possibility of changing business partners and reduce competitive flexibility.	Locking in long-term contracts			
The ability of some competitors to supply higher quality or more efficient drugs displaces demand and weakens the	Competitors' Advantage in Quality or Efficiency			

position of other companies.				
Replacing the drug with new technology or changing the production line to match the standards of another supplier imposes heavy costs on the company.	High Cost of Changing Production Technology			
The requirement to comply with GMP standards and international quality approvals makes the process of changing suppliers difficult and costly.	Quality and Safety Considerations			
The intensity of competition causes severe fluctuations in the amount of sales of companies and threatens the stability of operations.	Sales fluctuations	Income and liquidity instability	Consequences	
Competitive pressures lead to a drop in profit margins and a weakening of the cash flow of pharmaceutical companies.	Decrease in profitability			
Market instability and unpredictability of demand pose a serious challenge to production planning and supply of raw materials.	Disruption in production and supply planning			
The decline in profitability and liquidity pressure lead companies to borrow more from the banking system.	Increase in bank debt			

Fierce competition and lower-than-expected sales are causing drug depots in warehouses and increasing storage costs.	Inventory Accumulation and Warehousing Costs	Inefficiency of operations and production		
Financial pressure from competition reduces R &D budgets and limits companies' ability to innovate.	Reducing investment in research and development	Weakening the ability to develop and competitiveness		
Fierce competition makes companies incur more costs for advertising, discounts, and distribution network.	Increased marketing and logistics costs			
Competitive pressures and reduced profit margins can lead to a drop in drug quality or a shortage in the market.	Loss of quality and product shortages	Weakening production quality and damage to organizational credibility		
Marketing violations and opaque advertising expose companies to the risk of lawsuits and damage to brand reputation.	Legal and Credit Implications			
Pharmaceutical in the supply of raw materials or the distribution of products to reduce costs and avoid destructive competition.	Strategic alliances in production and distribution	Market Development and Differentiation Strategies	Controls & Responses	
Focusing on building a strong brand and promoting science instead of hefty discounts shifts the competition from price to quality and credibility.	Investing in Branding and Scientific Credibility			

Entering new therapeutic fields or producing less competing drugs reduces the intensity of direct competition.	Diversifying product portfolios			
Designing tenders in such a way that several companies win prevents the elimination of competitors and reduces unhealthy competition.	Multi-Winner Bidding Strategy	Strategies for Managing Competition in the Drug Market		
Market segmentation based on therapeutic, geographical, or price groups to reduce the intensity of disruptive competition and optimize market share.	Market segmentation			
Setting a stop loss and avoiding participating in auctions and tenders that threaten the long-term profitability of the company.	Conscious withdrawal from unprofitable tenders			
Expanding its operations into new markets, attracting different customer groups, and expanding its presence in different geographies such as Afghanistan and Iraq to reduce competitive pressure.	Diversifying markets and customers			
Facilitating and accelerating the regulatory and registration processes of drugs in order to reach the market faster and avoid falling behind in the competition.	Accelerating the process of registering new products	Innovation Strategies		

Making continuous improvements in formulation, packaging and manufacturing process to maintain a competitive advantage in a saturated market.	Incremental innovations			
Utilizing Lean and supply chain optimization approaches to reduce costs and increase operational efficiency.	Process Improvement and Cost Leadership	Cost Optimization and Value Creation Strategies		
Concluding contracts with insurance companies and medical centers in which payment depends on clinical effectiveness and treatment outcomes.	Value-based and result-based contracts			
Using Demand Elasticity Analysis and Consumer Behavior to Determine the Optimal Price and Maximize Profit in Competitive Conditions.	Intelligent Price Management Based on Demand Elasticity			
Assign parts such as distribution or packaging to specialized contractors to reduce fixed costs and focus on core activities.	Outsourcing non-key activities			
● Using innovative technologies (automation, ERP, business intelligence) to increase productivity and reduce operational costs.	Digitization of processes			
Collaborate with insurers to design co-curated treatment packages that reduce cost and increase effectiveness.	Managing the Shared Value Chain with Insurance			

Establish internal standards to prevent opaque payments, gifts, or unprofessional relationships with physicians and stakeholders.	Observance of anti-induction frameworks	Promoting Organizational Transparency and Credibility		
● Design and clear disclosure of pricing and discount policies to prevent misperceptions and unhealthy competition in the market.	Transparency in discount policies			

Proposition	Basic Code	Theme	Theme	Categories
The company is forced to split the profits in full because the government shareholder wants cash, but the result is a lack of liquidity to pay off the debts.	Obligation to distribute high dividends under shareholder pressure	Dividend policy and cash withdrawal from the company	Drivers / Policy Environment	Not to take risks
	Cash withdrawal from the company instead of accumulation for working capital			
	Weakness of Liquidity Conservation Policies in Pharmaceutical Holdings			
The government is raising the price of medicine with great delay, but the cost of	Order Pricing and Delay in Revision of Sales Rates	Policy and regulatory		

raw materials jumps every month, the profit margin disappears, and the ability to repay decreases.		pressure on profit margins		
	Sharp increase in the prices of raw materials and the exchange rate			
	Tax or insurance penalties that put pressure on liquidity			
	Hard limits for leverage and DSCR [16] in contracts	Contractual Restrictions and Banking Restrictions		
	Delay penalty and cross-default [17] clause activation between contracts			
	Requiring Frequent and Heavy Financial Reports for Banks			
	High share of non-cash items in unrealistic accounting profits	Low Earnings Quality and High Liquidity Cycle Weakness		
	Increasing the period of collection of receivables and warehouse inventory			
	Pressure on operating cash flow and inability to repay debt			

[16] Debt Service Coverage Ratio
[17] Cross-Border Patrol or Cross-Sectional Offense

If the bank decides not to renew, there is practically no other source; the company will face default.	Dependence on a bank or limited funding channel	Lender Concentration and Refinancing Risk		
	Non-renewal of credit lines due to restrictions of banks			
	Lack of an alternative option in the capital market or debt instruments			
When banks default, they take on a collateral asset that is practically not liquidated, and its value is half of the value of the books .	The market value of collateral is less than the balance of the debt	Inadequate collateral coverage and low liquidity of assets		
	Low liquidity of collateralized assets			
We took a foreign currency loan, but the income is in rials, and with this jump in the dollar, the debt doubled.	Foreign exchange financing for Rial projects	Foreign exchange debt with Rial income		
	Exchange rate jump and growth in the cost of repaying foreign exchange debt			
	Restrictions on foreign currency transfers due to sanctions			

They took a one-year loan for the three-year project, which means a definitive default; until then, production has not started yet, but the installments must be paid.	Short-term financing for long-term assets or projects	Mismatch of assets and liabilities		
	Inability to renew short-term lines of credit			
	Tight payback pressure in limited periods			
All debts are due in one period, banks don't renew either, you just have to choose which loan to default on.	Accumulation of maturing debts in less than a year	Focus on maturity and short-term debt wall		
	Lack of a redistribution plan or renewal facilitation			
	Increasing the risk of group default in the banking network			
The financial leverage is so high that with a small price shock, the company is out of the positive liquidity range.	High Net Debt to EBITDA Ratio	High financial leverage and fragility of capital structure		
	Increasing the share of short-term debt relative to total capital			

	Reduced ability to generate cash flow to cover interest and installments			
The medicine is sold, but the money comes from the insurance after a few months, until then we have to get a new loan to pay the installments	Increasing the period of collection of receivables and warehouse inventory	Liquidity pressure and increased need for working capital	Transmission channels to the firm	
	Obligation to make a cash advance payment for the import of raw materials			
	Locking up resources in requisition accounts and warehousing			
The bank said that the value of the collateral has decreased, you have to give additional collateral or settle part of the debt, the cash we had for production went to the bank	Depreciation of real estate or stock exchange collateral	Call for collateral and increased guarantee requirements		
	The bank's request to increase the collateral or convert it in cash			
	Compulsory sale of assets to maintain obligations			
When the dollar doubled, foreign currency installments became practically	Increase in the exchange rate while	Currency inequality in debts		

unpayable, and even the operating bank said that it was not possible to renew.	the company's income is Rials			
	Restrictions on access to currency and international transfers			
	Growth in the Rial Load of Foreign Currency Installments and the Risk of Default in Foreign Loans			
We took out a floating loan, and its rate went from 18 percent to 30 percent; only the interest swallowed half of the company's profits.	Growth of the effective rate of floating debts	Jumping interest rates and rising debt costs		
	Periodic reset of interest rates in banking contracts			
	The Effect of Contractionary Monetary Policies on the Financial Cost of Firms			
Last month, three of the company's facilities matured, but the bank did not take any action to renew them. As a result, the company's cash flow was suddenly disrupted and	Concentration of maturity of several loans in a limited time frame	Compression of due dates and short-term debt wall		

liquidity was severely reduced.				
	Lack of a debt restructuring or restructuring program			
	Sudden pressure on the company's liquidity in certain months			
Banks say they have no resources, and the bond market is closed, which means that there is practically no way to refinance	Non-renewal of loan lines due to banks' strictness	Refinancing Risk and Credit Window Closure		
	Lack of Alternative Instruments in the Capital Market			
	Decline in banks' confidence after delay or decline in domestic ranking			
The delay in financial reporting caused the bank to activate Trigger Covenant, and interest rates and penalties went up.	Exceeding the limits of financial leverage or specified in the contract	Breach of contractual obligations and activation of default clauses		
	Delay in submission of periodic financial reports			
	Cross-Default or Cross-Penalty Rate Activation			

Operating cash doesn't answer the financial cost; we have to use a new loan to pay off the previous loan	Reduced operating cash flow compared to the cost of debt	Decline in debt servicing capacity and weak operating cash flow		
	Decrease in sales or increase in the cost of production compared to the forecast			
	Poor ICR ratio			
When there is no longer any renewal, the company will either have to sell the asset or move to financial restructuring; it is not possible to continue with the current structure.	Entering the process of dissolution or reorganization of the company	Bankruptcy and financial restructuring	Consequences	
When the accumulation of debts exceeds a threshold, the pharmaceutical company has no choice but to implement emergency settlement or merger plans, which are taken not by choice, but to prevent default and maintain a minimum of survival.	Applying emergency settlement or integration plans for survival			
	Selling assets to repay debts			
After the default, even the foreign supplier was not	Loss of trust of banks and suppliers			

willing to pre-sell; he said to do the previous settlement first.				
	Cancellation or modification of contracts by commercial parties	Credit and Reputational Damage		
	Decline in domestic rank and deprivation of some auctions and tenders			
The occurrence of an initial default led to an increase in the interest rate of the facility, which consequently put more pressure on the company's cash flow. As a result, the company's liquidity situation deteriorated, and several other consecutive defaults followed.	Increased financial cost after default	Exacerbation of liquidity risk and the domino default cycle		
	Deterioration of the Cash Cycle Index and the Decline of Free Cash			
	The Rise of Chain Failures in Corporate Governance or Distribution Companies			
The bank did not pay, the supplier did not send the materials, the production line slept for two weeks	Shortage of raw materials and parts due to the	Disruption of operating cash flow and		

and the market was empty	discontinuation of suppliers' credit	continuity of production		
	Decrease in market coverage and sales share			
	Stopping production lines or reducing operating capacity			
When liquidity is spent solely on repaying debt, there is no money left for innovation or new design.	Postponement or cancellation of R&D projects	Reduction in investment and development capacity		
	Stopping equipment and technology upgrade plans			
	Reducing investment in the production of new products			
The banks said that since you defaulted, we will not open any other credit, now we only buy and sell cash.	Reducing the ceiling of banking lines and new credits	Restricted access to credit resources		
	Increasing bail conditions and delay penalty			
	Removal or suspension of credit by broadcasters and insurances			
The bank confiscated the machinery, that is, in order to pay the debt, the means of	Seizure or Sale of Manufactured Assets by Banks	Seizure of collateral and		

production itself were destroyed		forced sale of assets		
	Loss of productive assets such as production lines or land			
	Weakening operational capacity in the long term			
One default caused the interest rate to go from 24 percent to 32 percent, and from then on, the company's profits became almost zero	Increasing the effective interest rate after default	Increased cost of debt and financial penalties		
	Penalty for Late Payment of Installments			
	Growth in financial costs and decrease in net profit			
When a liquidity crisis becomes serious, the company quickly forms a crisis team to meet with creditors and write a quarterly cash plan.	Formation of a financial revival working group with the presence of the CEO and the financial manager	Financial and managerial restructuring	Controls & Responses	
	Appointing an independent restructuring manager to negotiate with creditors			

	Designing a 100-day cash plan to overcome the crisis[18]			
When the bank sees that the collateral has become more real or the losing part has been separated from the company, it usually shows more support.	Replacement of illiquid collateral with productive assets	Optimization of collateral and legal structure		
	Separation of healthy assets from loss-making to restructure the group structure			
	Using physical assets to increase collateral coverage			
With the interim agreement, the pressure from the creditors stops and the company gets a breathtaking opportunity to restructure the debt.	Signing Standstill Agreement with Major Creditors	Coordination and negotiation with creditors		
	Formation of a Coordination Committee between Banks and Financial Institutions			
	Appointing a Joint Representative to Manage Credit Negotiations			

[18] The "100-day cash plan" refers to a short-term, intensive plan to manage cash flow, reduce unnecessary expenses, and maintain the ability to pay the company's vital obligations within a period of about three months.

In some cases, we were able to settle debts in cash with a 10 to 15 percent discount and escape official default.	Redeeming part of the debt at a discount	Active Debt Management		
	Converting Short-Term Loans to Long-Term Loans			
	Debt Replacement Proposal			
We had to sell a shed, but we rented it so that production would not sleep and liquidity would be released.	Sale of land or inactive warehouses to free up cash	Release of liquidity from assets		
	Execution of Sale & Leaseback on Manufacturing Machinery			
	Transfer of Non-Operating Assets to Investment Funds			
	Cash injection of major shareholders	Capital Strengthening and Improving Financial Leverage		
With the conversion of debt to equity, the debt ratio has decreased, and banks have regained confidence to extend the credit line.	Converting part of the debt into shares of the company			
	Raising capital from venture capital and development funds			

We took out a short-term loan just to get through the liquidity crisis, the interest rate was high, but it was a lifesaver.	Use of Bridge Financing [19]	Alternative and flexible financing		
	Receiving Mezzanine facilities at a higher rate but for a shorter period			
	Backstop Contract with Bank for Emergency Injection[20]			
With continuous negotiations, we were able to delay the installments by three months so that the new sale would cover the debt.	Extending the Debt Ceiling by Negotiations	Renewal and revision of debt terms		
	Installment Breathing Cycle Agreement			
	Reducing effective interest rates through re-agreement with the bank			
In the crisis situation, we went to drugs that pay faster because we were not looking for profit, we were looking for survival.	Negotiations with the Food and Drug Administration to reform the price of medicines	Price, sales and cost management		

[19] Bridge Loan (Bridge Financing) means short-term financing used to bridge the gap between two sources of liquidity, such as a bridge that moves the company from its current situation to more stable sources.
[20] Backstop Contract: A liquidity backup contract refers to a reassuring agreement with a bank or investor that aims to guarantee an injection of funds in an emergency. That is, when the company's other sources of cash are unavailable or markets are closed.

	Reduced administrative and non-operational costs			
	Manage the sales mix with a focus on more liquid products			
Only by reforming the cash cycle was we able to free up 20% of liquidity without new loans	Reducing DSO through Follow-up of Insurance Claims Collection	Improve the cash conversion cycle		
	Increasing DPO through Supplier Negotiation			
	Reduce DIO by controlling warehouse levels and preventing accumulation			
When the alert system was working properly, we would notice the crisis before the installment fell behind	Daily monitoring of DSCR, ICR and CCC indicators	Early Warning System		
	Designing a Warning Dashboard for Liquidity Risks			
We would send financial reports ahead of time so that the bank would feel that the situation was under control and we would take the obligations seriously.	Headroom Monitoring in Financial Ratios	Covenant Management & Reporting		

	Waiver Playbook Design for Asking Banks for Deadlines			
	Strengthening financial reporting and transparency in disclosure			
With the diversity of banks, if one of them closed its line, we used the others and the liquidity was not cut off.	Negotiating with multiple banks instead of one source	Diversification of financial resources		
	Use of non-bank financing instruments such as bonds			
	Entering the Pharmaceutical Industry Funds for Special Facilities			
	IRS, Cap/Floor Use	Hedging Interest Rate and Exchange Risk		
	Currency Hedging for Dollar or Half Debts			
	Creation of a Debt Service Reserve Account (DSRA)	Cash Reserves and Pledged Lines		
	Credit lines ready for emergencies			
When we set a debt ceiling for ourselves, we did not cross the danger point even without warning the bank.	Definition of Domestic Net-Debt/EBITDA Ceiling	Leverage Policy and Debt Limits		

	Determination of DSCR and ICR Minimum for Internal Alert			
Now the debt maturities are spread out throughout the year, so we no longer have a seasonal cash crisis.	Laddering of Debt Installments	Alignment of due dates and debt ladder		
	Matching the maturity of debts with incoming cash flow			
If insurance companies make payments on time, 80 percent of pharmaceutical companies' default problems will be solved.	Requiring insurers to pay claims on time	Coordination with the government and insurance		
	Pursuing government commitments in the form of multilateral agreements			

Proposition	Basic Code	Theme	Theme	Categories
Companies that do not have product diversification will suffer a financial shock with any change in foreign exchange policy or insurance circulars.	Small-scale companies and a strong focus on one or more products	Limited business size and variety	Drivers / Policy Environment	Credit Rating Change Risk

	Dependence on a few customers or a specific market			
	High sensitivity to macroeconomic shocks or currency fluctuations			
Most companies only work with one bank; when that bank gets tough, the refinancing is effectively locked.	The company's dependence on a bank or limited financing channel	Concentration of Financing and Refinancing Risk		
	The Wall of Darkness (Short Story)			
	Lack of access to capital market tools for debt renewal			
Financial disclosures are delayed, decisions are arbitrary, and government policies do not allow companies to have sustainable financial planning.	Delay in Financial Disclosure and Reporting	Weakness of Corporate Governance and the Regulatory Environment		
	Order pricing policies that make profitability unpredictable			
	Lack of transparency and professional decision-making system at the board level			
When we took out a foreign currency loan, the dollar tripled, and both the principal of the loan and the	Uncovered foreign exchange debts	Currency Risks and Floating Interest Rates		

floating interest rate of the rial increased .				
	Increase in the cost of financing due to periodic interest rate resets			
	Real interest rate fluctuations in the banking system			
	Increase in non-cash items in profits (unrealistic EBITDA[21])	Poor quality of profits and liquidity		
	Unstable and negative operating cash flow in consecutive periods			
	Increase high cash conversion cycles			
The leverage is so high that with a small change in interest rates or sales, the DSCR becomes negative.	High debt-to-interest ratio	High financial leverage and inadequate debt coverage		
	Weakness in the Low Debt Service Coverage Index (DSCR[22])			
	Rapid growth of short-term debt against cash assets			
In Iran, credit risk does not make sense, because everyone borrows at the same	Lack of a reputable credit rating institution in Iran			

[21] Earnings Before Interest, Taxes, Depreciation and Mortization
Earnings Before Interest, Taxes, Depreciation and Impairment of Assets
[22] Debt Service Coverage Ratio
Debt Repayment Coverage Ratio

rate, there is no rank, and there is no difference between good and bad accounts		Institutional weakness and lack of a credit rating system		
	Interest rates are unrelated to credit risk			
	Orderly control of banks' balance sheets			
	Lending to a Single Beneficiary in Pharmaceutical Holdings			
We had an unsuccessful GMP inspection , production stopped, and in the same month our credit rating dropped because the financial statements were locked	Issuing heavy judicial or tax rulings against the company	Legal and operational events with a credit effect	Transmission channels to the firm	
	Food and Drug Administration penalties for GMP/GDP defects			
	Downtime of production lines due to quality accident or failed inspection			
	Negative media coverage of drug violations or poor quality			
When banks don't have resources or the bond market is closed , even well-performing companies can't renew their previous debt.	Difficulty in renewing bank credit lines due to regulatory ceilings	The risk of refinancing and closing the market window		

	Increase in the cost of new financing in the bond market			
	Limitation of access to the debt market during periods of currency or policy shocks			
Because we delayed the financial statement, the bank activated the Quick Trigger Covenant and the credit rating went down.	Bypassing the ratios committed in financial contracts, such asdebt/EBITDA	Violations of Covenants and Delays in Financial Reporting		
	Delay in sending financial statements or disclosures to the stock exchange and banks			
	Lowering Headroom and Activating Downgrade Triggers			
	Failure to fulfill key performance indicators in sales or profitability	Deviation from Rank Guide and Performance Gap		
	Distancing Actual Performance from Ranking Model Assumptions			
	Decrease in the ratio of cash coverage to net profit to liabilities			
After several delays in repayment, the bank placed us on the special watch list and the renewal of credits was stopped.	The company is placed on a special watch list or Watch Placement	Change of perspective or special supervision		

	Change Outlook from stable to negative or degraded			
	Increasing the frequency of monitoring by the rating body or the Central Bank			
When the rating goes down, the supplier loses its trust, the money comes later, liquidity locks in, and projects go to sleep.	Creating a vicious cycle of liquidity	Contagion to other risks and liquidity freeze	Consequences	
	Suspension or Postponement of Investment Projects			
	Intensifying Chain Effects on Production, Supply and Credit			
After the drop in rank, even the old broadcasters made a prepayment bet, and the company's commercial reputation was damaged.	Reducing the credit limit by broadcasters and major buyers	The Business Domino Effect and Brand Damage		
	Pessimism in the market about the company's ability to pay			
	Hardening the collection of receivables and increasing the period of collection of receivables			
The bank said that since your rating has dropped, you should	Increasing the collateral-to-loan ratio			

put collateral again; practically half of our liquidity was blocked.		Increase in collateral demand and stricter conditions by banks		
	Obligation to pay cash advance in contracts			
	Applying Margin Call or Additional Collateral on Current Facility			
When the rating went down, the banks did not renew, we have to take new loans at a much higher rate	Reduction or elimination of bank lines of credit	Reduced access to financial resources and lines of credit		
	Reducing the ceiling of commitments and extending with stricter terms			
	Lack of access to the debt market or new securities			
As the rating dropped, the interest on the auto loan went up, the DSCR fell below one, and the company had to roll back some instalments.	Increasing the effective interest rate	Rising debt costs and pressure on financial indicators		
	Decline in Debt Service Coverage Index			
	Activation of Step-up Clause in Contracts			
When we saw the signs of a downgrade, we quickly went to negotiate with the banks, sold some of the assets and	Optimizing collateral and assets to maintain credit lines	Corrective Actions in Situations Subject to Demotion	Controls & Responses	

distributed the debt maturities so that the maturity wall would be broken.				
	Use alternative sources of cash such as bridge financing or short-term lines of support			
	Negotiating to extend maturities or adjust interest rates and remove the maturity wall			
	Reducing Leverage Rapidly (Deleveraging) through Asset Sales, Capital Raising, or D/E Swap			
We publish a stakeholder communication report for each quarter so that banks know that our cash flow is stable, because transparency is the best shield against downgrade.	Transparent disclosure and active communication with financial institutions to manage market expectations	Rank Strategy and Prevention of Decline		
	Improving the Liquidity Cycle and Profit Quality			
	Covering interest rate and currency fluctuations[23] to prevent credit shocks			
	Diversification of financial resources			

[23] *(IR/FX Hedging)*

	and design of the maturity ladder[24]			
	Prudent Leverage and Liquidity Policies			
	Defining and monitoring the ranking scenario and determining the KPIs related to maintaining the rank			
Proposition	Basic Code	Theme	Theme	Categories
We don't have any reliable sources to check the credibility of the other party, some companies are healthy on paper, but behind a set of debts or opaque ones.	Weakness in Corporate Governance Structure and Lack of KYC[25]/KYB Database[26]	Weak Corporate Governance and Information Transparency	Drivers / Policy Environment	Counterparty risk
	Ambiguity in Firms' Ownership and Conflicts of Interest in Business Relations			
	Lack of a transparent and centralized accreditation system for industry activists			
When the government budget arrives late or the currency changes, broadcasters and hospitals are the first places their payments are locked.	Risk from Sanctions and International Settlement Restrictions	Macroeconomic Risks and Regulatory Constraints		

[24] *Maturity Ladder*
[25] Know Your Customer
[26] Know Your Business

	Focus of companies' portfolios on a few large buyers or suppliers (India and China)			
	Inflationary pressure and exchange rate fluctuations on the other side's financial ability			
	Delay in allocating government budgets and poor implementation of contracts			
Our tool is just a check, not a credit report, not a risk score. Even a formal contract sometimes doesn't exist.	Reliance on traditional guarantees (checks, promissory notes, bank guarantees)	Weaknesses in Credit Control and Evaluation Tools		
	Evaluation based on personal knowledge or old relationships rather than financial indicators			
	Starting a limited volume of cooperation to reduce the risk of newly established companies			
If one of the distributors suffers from a cash crisis, the entire chain from production to pharmacy will be locked, and we	Longevity of the supply chain and the interdependence of liquidity between the rings	Chain Risks and Cash Codependence		

practically have the risk of chain default.				
	High default power in distribution companies or intermediate buyers			
	The Impact of a Loop's Financial Behavior on the Entire Payment Chain			
The company's claims from insurance companies and distribution companies are transferred to financial institutions to transfer the risk of default. Although this method requires the payment of fees or fees, it is considered as a more desirable way to prevent liquidity from being locked.	Using Discounting Claims (Factoring) to Reduce the Risk of Default	Financial Responses and Tools to Reduce Exposure		
	Issuance of debt securities to provide cash and control the pressure of settlement delay			
Concentrating payments in a bank is a big risk; if that bank goes into trouble, the settlements will be locked and the company's liquidity will stop.	Centralization of transactions in one or two state-owned or sanctioned banks	Banking & Financial Risk	Transmission channels to the firm	

	Delays in transferring funds due to interbank or international settlement problems			
	Restrictions on the Use of Financial Instruments (LCs), Guarantees, Factoring			
Whenever the insurance companies run a budget deficit, they do not delay payments , which means that our entire liquidity is under pressure.	Delay of several months for insurers in verifying and paying claims	Risk of Insurance Policies and Institutional Payers		
	A sudden change in the insurance coverage of medicines			
	Reducing the share paid by insurance and increasing the company's cash burden			
Some drug distribution companies settle the funds from the sale late or incompletely, and due to the lack of an effective regulatory mechanism, the manufacturing company is not able to control this process.	Deviation in the performance of distribution companies and sales representatives	Intermediary Risk & Agency[27]		

[27] Agent Risk

	Incomplete or non-transparent reporting by intermediaries			
	Unethical Behavior or Abuse of Representative Position			
	Violation of the terms of the contract by the customer or supplier	Contractual and Legal Risk		
	Disputes in the interpretation of payment and settlement clauses			
There is a contract, but it does not have an enforcement guarantee; if the other party does not fulfill its commitment, it will take a year for you to file a complaint.	Lack of a strong executive guarantee for the implementation of financial obligations			
	Delay in interbank clearing	Bank Settlement Risk and Funds Transfer		
	Blocking of funds in the international transfer route			
	Systemic errors or settlement errors in accounting			
If the foreign supplier does not send the goods on time, we will lose our obligations, which means that	Delay in the delivery of imported raw materials or medicines	Supplier Functional Risk		

liquidity will also disappear and the trust of the market will also be lost.				
	Poor quality of the shipment or non-compliance with the standard			
	Dependence on a limited number of key suppliers			
Broadcasters usually settle with long delays; sometimes even a portion of the money never returns, only goes to the account of burned claims.	Default or delay in payment by distributors and pharmacies	Customer Credit Risk		
	Lack of valid collateral or warranty for new customers			
	Increase in suspicious claims on the balance sheet			
When the outstanding claims increase, the banks no longer trust; for each guarantee, we have to put a multiplier for each guarantee.	Damage to the company's reputation against banks and suppliers	Financial pressure and declining business credit	Consequences	
	Tightening of the conditions for obtaining facilities or guarantees			
	Reducing access to credit lines or			

	increasing effective interest rates			
	The need to use costly settlement methods such as LC, Screw, [28]or Bank Guarantee	Increased settlement costs and secure financial requirements		
	Reduced flexibility in financial relationships with customers			
	Growth in bank fees and collateralization costs			
When a supplier defaults or doesn't ship materials, the production line goes dormant and competitors quickly take market share	Stoppage of production lines due to supplier default or delay	Interruption in supply and production		
	Delay in the delivery of raw materials or intermediate drugs			
	Loss of market share due to lack of supply			
Because the demands come late, we have to hold more cash; in fact, the CCC has become more negative and the cash pressure is greater than ever.	Increase in the duration of collection of receivables	Increasing need for working capital and worsening cash cycle (CCC)		

[28] Escrow is like an "escrow account" in which the money of the transaction is blocked until the work is done.

	Requiring a cash advance for new purchases			
	Growing dependence on short-term loans and increasing financial costs			
In order to receive a six-month check, you have to start for a year, and at the end half of it will be returned, and the other half will go to the fuel of the claims.	Increase in suspicious claims and fuel claims	Direct losses from outstanding claims and legal fees		
	Litigation Fees and Legal Follow-up for Collecting Claims			
	Spending time and resources to resolve contractual disputes			
We have a contract model for the government, another model for the private sector. We take a down payment with the public hospital, but we set a short settlement deadline for private distribution.	Defining different contractual terms for the public and private sectors	Contract Management and Market Channel Separation	**Controls & Responses**	
	Applying credit limits, down payments, or guarantees to high-risk clients			

	Creating sales policies tailored to the credibility of each distribution channel			
We will no longer allow sales to be in the hands of only a few specific broadcasters, because if one of them defaults, the entire sale will fall asleep.	Reduced dependence on multiple buyers or large spreads (lower HHI index)	Diversification of business parties and reduced credit concentration		
	Expanding the network of smaller customers and suppliers to distribute risk			
	Continuous evaluation of sales share to key customers			
Now, we have put a netting clause in the contracts so that if demand and debt are at the same time, we can barter and release cash faster.	Predicting Netting and Set-off Mechanism for Mutual Debt and Demand Settlement	Improving contractual provisions and the use of protective clauses		
	Definition of Arbitration and Dispute Resolution Clauses (Arbitration, ICC, Step-in Rights)			
	Setting a cap for the amount of chargeback and rebate in contracts			
We put SLAs in contracts and late fines, which has caused the other party	Insertion of SLA (Service Level Agreement) and Contract Delay Penalty			

to be more disciplined in payments.		Promotion of contractual discipline and performance monitoring		
	Obligation for periodic financial reporting by the other party			
	Determining Performance Indicators for Evaluating Payment and Collaboration Behavior			
